# Supplementary material for: Inhibiting MARSs reduces hyperhomocysteinemia‐associated neural tube and congenital heart defects
Source: EMBO Mol Med. 2020 Jan 31;12(3):e9469. doi: 10.15252/emmm.201809469 (PMC7059139; doi:10.15252/emmm.201809469)
Supplement: Supplementary file 1 — Appendix [file EMMM-12-e9469-s001.pdf]

Appendix (Xinyu Mei et al)

Appendix Tables:

Appendix Table S1. NTD and CHD samples used in this study
Appendix Table S2. Primers used to quantitate MARS and MARS2 copy numbers by AccuCopy assay and qPCR in this study
Appendix Table S3. Cell lines used in this study
Appendix Table S4. Details of antibodies used in this study
Appendix Table S5. Real-time PCR primers used to quantify mRNA expression
Appendix Table S6. Recombinant DNAs used in this study
Appendix Table S7. List of P-values

Appendix Figures:

Appendix Figure S1. MARS expression in colorectal cancer and adjacent non-tumour tissue
Appendix Figure S2. Strategy for the cell-wide proteomic survey for N-Hcy substrates
Appendix Figure S3. MS/MS spectra of lysine residues that are N-Hcy modified
Appendix Figure S4. AHT decreased N-Hcy and β-catenin levels

Appendix Tables:

Appendix Table S1. NTD and CHD samples used in this study

**196 neural tube defects**	
Craniorachischisis	59
Open Spina bifida	38
Anencephaly plus Open cervical spina bifida	38
Meningoencephalocele	5
Anencephaly plus Open thoracolumbosacral	5
Meningoencephalocele plus Open lumbosacral spina bifida	6
Closed spina bifida	10
Myelomeningocele	4
Anencephaly	4
Anencephaly plus Meningoencephalocele	27
**100 congenital heart defects**	
Ventricular septal defect (VSD)	49
Atrial septal defect (ASD)	21
Ventricular and septal defect (VASD)	10
Patent ductus arteriosus	16
Transposition of great arteries (TGA)	4
a control group of 240 cases	

CHDs and NTDs samples were collected in Capital Institute of Pediatrics during 2010-2015 regardless the Hcy and HTL levels of the mothers.

Appendix Table S2. Primers used to quantitate copy numbers of MARS and MARS2 by AccuCopy assay and Q-PCR in this study

Primers	Sequence	Purpose
Accu-MARS-F1	AGCGACAGAGCTGCAGGTAGGA	Detect MARS in AccuCopy
Accu-MARS-R1	TGTTCTTGGTTCAGTGCGGAGA	Detect MARS in AccuCopy
Accu-MARS-F2	GAGCAACTGCGATGTGAGCACT	Detect MARS in AccuCopy
Accu-MARS-R2	GTGTGGCAAGCTCATCAATGCT	Detect MARS in AccuCopy
Accu-MARS-F3	AGCCAAGCCACAGCAGATACAA	Detect MARS in AccuCopy
Accu-MARS-R3	GCACAAAAGGCAGACAAGAACG	Detect MARS in AccuCopy
Accu-MARS2-F1	CAAGGGCGTCTATGAAGGTTGG	Detect MARS2 in AccuCopy
Accu-MARS2-R1	CATCGGGGGATTCGTTTCCT	Detect MARS2 in AccuCopy
Accu-MARS2-F2	GGACATCCATGCCCTTTTGAAG	Detect MARS2 in AccuCopy
Accu-MARS2-R2	AAAGCCCACCGGACCTAGAAAC	Detect MARS2 in AccuCopy
Accu-2p-F1	GCCAAAAATTCAGAATACAAGGA	Reference in AccuCopy
Accu-2p-R1	GTTTGCCTGCCTTCCAAGCAA	Reference in AccuCopy
Accu-10pL-F1	CACTGAGCCCCAGAGACCTGAC	Reference in AccuCopy
Accu-10pL-R1	GTTTTCCCTGGAGGTGTGCATT	Reference in AccuCopy
Accu-20q-F1	AGGGTGCTGGGATCAGAGAGAG	Reference in AccuCopy
Accu-20q-R1	CATTTTGCCACCCTCCAGTAGC	Reference in AccuCopy
qPCR-MARS-F1	TGGAATGGGAAGCGACAGA	Reference in qPCR
qPCR-MARS-R1	AGCAGACAAAGCTGGCTAAAATG	Reference in qPCR
qPCR-MARS-F2	GTATCCTGCTGACAAACTTCCTG	Reference in qPCR
qPCR-MARS-R2	GGAGTCCCATGACCAAAGTAAC	Reference in qPCR
qPCR-MARS2-F1	GGATCCTAGGACTTGCCTTAACC	Reference in qPCR
qPCR-MARS2-R1	TCATAGTAGTCACAGTCCCAGTT	Reference in qPCR
qPCR-MARS2-F2	GGACCTGAAACTGGGCTTTTG	Reference in qPCR
qPCR-MARS2-R2	CCACAAGCCGGTAAGAACTGA	Reference in qPCR
qPCR-MTR-F1	ACCTTCCTGAAGCCCACTTG	Reference in qPCR
qPCR-MTR-R1	CCTGCCTCGTCTTACTGTCC	Reference in qPCR
qPCR-IDH1-F1	AAAGTCGAAGCAGTTGGAAGTT	Reference in qPCR

Appendix Table S3. Cell lines used in this study

Cell Lines		
NE-4C	ATCC	Number: CRL-11268
H9C2	ATCC	Number: CRL-1446
HEK293T	ATCC	Number: CRL-2925

Appendix Table S4. Details of antibodies used in this study

Antibodies			
Mouse anti SOD1	Santa Cruz	sc-8637	1:1000
Mouse anti SOD2	Abcam	ab16954	1:1000
Mouse anti β-catenin	Abcam	ab22656	1:1000
Rabbit anti MARS	Abcam	ab180497	1:1000
Rabbit anti MARS2	Abcam	ab130426	1:1000
Mouse anti MARS	Abcam	ab50793	1:500
Rabbit-Anti-ki67	Abcam	ab15580	1:500
Rabbit-Anti-Ki67	Sigma	SAB5500134	1:500
Mouse-anti-Tuj1	Covance	MMS-435P	1:500
Rabbit anti Flag	Cell Signaling Technology	14793S	1:1000
Mouse anti β-actin	Cell Signaling Technology	3700	1:5000
Rabbit anti Flag	Abmart	AB_2713960	1:1000
Mouse anti Myc	Abmart	Cat#M20002	1:1000
anti-mouse secondary antibodies	GenScript	AB_1968937	1:5000
anti-rabbit secondary antibodies	GenScript	AB_1968815	1:3000

Appendix Table S5. Realtime-PCR primers used to quantify gene expression.

Realtime-PCR Primers	
c-Myc-F	CTTCTCTCCGTCCTCGGATTCT
c-Myc-R	GAAGGTGATCCAGACTCTGACCTT
cyclinD1-F	CATCAAGTGTGACCCGGACTG
cyclinD1-R	CCTCCTCCTCAGTGGCCTTG
GAPDH-F	CTCTTCCACCTTCGATGCC
GAPDH-R	GGGGAGATGCTAGTGCTCTTG

Appendix Table S6. Recombinant DNAs used in this study

Recombinant DNAs	Source
pRK7-Flag-MARS	In this paper
pRK7-Flag-MARS2	In this paper
pcDNA3.1-Flag-Bcl-2	A gift from lab of Dr. Jiahuai Han at Xiamen University, China.
pcDNA3.1-Flag-SOD1	In this paper
pcDNA3.1-Flag-SOD2	In this paper
pcDNA3.1-Myc-Dvl1	A gift from lab of Dr. Hongyan Wang at Fudan University, China.
pcDNA3.1-Flag-NRX	A gift from lab of Dr. Hiroaki Miki at University of Tokyo, Japan (Funato et al., 2006)
TOPFLASH	A gifted from Dr. Tao Zhong’s lab, Fudan University (Ni et al., 2011)
FOPFLASH	Millipore, Bedford, MA, USA

Appendix Table S7. List of P-values

Figure	Statistical Test	Comparison	p-value
Fig. 1A	Unpaired t test	2 copies vs. 3 or 4 copies	0.0262
Fig. 1B	Unpaired t test	2 copies vs. 3 or 4 copies	0.0202
Fig. 1D	Unpaired t test	2 copies vs. 3 or 4 copies	0.0048
Fig. 1F	Unpaired t test	2 copies vs. 3 or 4 copies	0.0053
Fig. 1K	Wilcoxon matched pairs test	D vs. C	0.0110
Fig. 1L	Wilcoxon matched pairs test	D vs. C	0.0078
Fig. 1M	Wilcoxon matched pairs test	M vs. F	0.0061
Fig. 1N	Wilcoxon matched pairs test	M vs. F	0.0092
Fig. 2C	Two way ANOVA	MARS-Hcy- vs. MARS-Hcy+	0.0124
		MARS+Hcy- vs. MARS+Hcy+	0.0001
Fig. 2D	Two way ANOVA	MARS2-Hcy- vs. MARS2-Hcy+	0.0049
		MARS2+Hcy- vs. MARS2+Hcy+	0.0001
Fig. 2E	One way ANOVA	Met0 vs. Met75	0.9984
		Met0 vs. Met100	0.9939
Fig. 2F	Two way ANOVA	MARSs-Hcy- vs. MARSs-Hcy+	0.0001
		MARSs+Hcy- vs. MARSs+Hcy+	0.0314
Fig. 3A	Unpaired t test	MARS- vs. MARS+(HTL)	0.0100
		MARS- vs. MARS+(Superoxide)	0.0054
Fig. 3B	Unpaired t test	MARS2- vs. MARS2+(HTL)	0.0117
		MARS2- vs. MARS2+(Superoxide)	0.0013
Fig. 3C	Unpaired t test	shMARS- vs. shMARS+(HTL)	<0.0001
		shMARS- vs. shMARS+(Superoxide)	<0.0001
Fig. 3D	Unpaired t test	shMARS2- vs. shMARS2+(HTL)	0.0160
		shMARS2- vs. shMARS2+(Superoxide)	0.0022
Fig. 3E	Two way ANOVA	WT Hcy-Met- vs. WT Hcy+Met-	<0.0001
		WT Hcy-Met- vs. WT Hcy-Met+	0.9996
		shMARSs Hcy-Met- vs. WT Hcy+Met-	>0.9999
		shMARSs Hcy-Met- vs. WT Hcy-Met+	>0.9999
Fig. 3F	One way ANOVA	Hcy0 vs. Hcy10	0.0004
		Hcy0 vs. Hcy20	<0.0001
Fig. 3G	Two way ANOVA	MARS-Hcy- vs. MARS- Hcy+	0.0100
		MARS+Hcy- vs. MARS+ Hcy+	<0.0001
Fig. 3K	Two way ANOVA	WT Hcy-HTL- vs. WT Hcy+HTL-	0.0003
		WT Hcy-HTL- vs. WT Hcy-HTL+	<0.0001
		shMARSs Hcy-HTL- vs. WT Hcy+HTL-	0.9639
		shMARSs Hcy-HTL- vs. WT Hcy-HTL+	0.0001
Fig. 4C	Two way ANOVA	WT HTL- vs. WT HTL+(SOD1)	<0.0001
		3KW HTL- vs. 3KW HTL+(SOD1)	0.4450

		WT HTL- vs. WT HTL+(SOD2)	<0.0001
		5KW HTL- vs. 5KW HTL+(SOD2)	0.2324
Fig. 4D	One way ANOVA	WT vs. K23W (SOD1)	<0.0001
		WT vs. K122W (SOD1)	<0.0001
		WT vs. K128W (SOD1)	<0.0001
		WT vs. 3KW (SOD1)	<0.0001
Fig. 4E	One way ANOVA	WT vs. K44W (SOD2)	<0.0001
		WT vs. K51W (SOD2)	0.0033
		WT vs. K98W (SOD2)	0.0005
		WT vs. K106W (SOD2)	0.0017
		WT vs. K178W (SOD2)	<0.0001
		WT vs. 5KW (SOD2)	<0.0001
Fig.4E	Unpaired t test	HTL- vs. HTL+ (SOD1)	0.0057
	Unpaired t test	HTL- vs. HTL+ (SOD2)	0.0091
Fig.4F	Unpaired t test	HTL- vs. HTL+ (SOD1)	0.0017
	Unpaired t test	HTL- vs. HTL+ (SOD2)	0.0099
Fig. 4G	Unpaired t test	shMARSs- vs. shMARSs+ (SOD1)	<0.0001
	Unpaired t test	shMARSs- vs. shMARSs+ (SOD2)	<0.0001
Fig. 5A	One way ANOVA	Hcy0 vs. Hcy10	0.0196
		Hcy0 vs. Hcy20	0.0001
		Hcy0 vs. Hcy50	<0.0001
Fig. 5B	One way ANOVA	HTL0 vs. HTL5	0.9927
		HTL0 vs. HTL10	0.0012
		HTL0 vs. HTL10	0.0001
Fig. 5C	Unpaired t test	Hcy- vs. Hcy+	<0.0001
	Unpaired t test	HTL- vs. HTL+	<0.0001
Fig. 5D	Two way ANOVA	Hcy-shMARS- vs. Hcy+shMARS-	0.0001
		Hcy-shMARS+ vs. Hcy+shMARS+	0.8918
Fig. 5E	One way ANOVA	Hcy0 vs. Hcy15	0.0496
		Hcy0 vs. Hcy25	<0.0001
		Hcy0 vs. Hcy50	<0.0001
Fig. 5F	One way ANOVA	Hcy0 vs. Hcy5	0.7097
		Hcy0 vs. Hcy10	0.0035
		Hcy0 vs. Hcy20	<0.0001
Fig. 5G	Two way ANOVA	Hcy-shMARS- vs. Hcy+shMARS+	0.0011
		Hcy-shMARS- vs. Hcy+shMARS+	0.9697
Fig. 5H	Two way ANOVA	Hcy-SODs- vs Hcy+SODs-	0.0001
		Hcy+SODs- vs Hcy+SODs+	0.0002
		HTL-SODs- vs HTL+SODs-	<0.0001
		HTL+SODs- vs HTL+SODs+	0.0064
Fig. 5I	Two way ANOVA	HTL-SODs- vs. HTL+SODs-	0.0002
		HTL-SOD+ vs. HTL+SODs+	0.8290
Fig. 6D	One way ANOVA	AHT0 vs. AHT10	0.0500

		AHT0 vs. AHT30	0.0094
		AHT0 vs. AHT70	0.0010
Fig. 6E	One way ANOVA	AHT0 vs. AHT70	0.2271
		AHT0 vs. AHT100	0.0301
Fig. 6F	Unpaired t test	Ctrl vs. AHT (NE4C)	0.3597
	Unpaired t test	Ctrl vs. AHT (H9C2)	0.8931
Fig. 6H	Two way ANOVA	AHT0 vs. AHT10	0.0087
		AHT0 vs. AHT30	<0.0001
		AHT0 vs. AHT70	<0.0001
Fig. 6I	Two way ANOVA	AHT0 vs. AHT10	0.0499
		AHT0 vs. AHT30	0.0063
		AHT0 vs. AHT70	0.0054
Fig. 6J	Two way ANOVA	Hcy-AHT- vs. Hcy+AHT+	0.0073
		Hcy-AHT+ vs. Hcy+AHT+	0.9816
Fig. 6K	Two way ANOVA	CtrlAHT- vs. HcyAHT-	0.0008
		CtrlAHT+ vs. HcyAHT+	0.9942
	Two way ANOVA	CtrlAHT- vs. HTLAHT-	<0.0001
		CtrlAHT+ vs. HTLAHT+	0.0001
Fig. 7B	One way ANOVA	Ctrl vs. ATRA (MARS)	0.0072
		ATRA vs. ATRA+AHT (MARS)	0.1542
	One way ANOVA	Ctrl vs. ATRA (N-Hcy)	0.0035
		ATRA vs. ATRA+AHT (N-Hcy)	0.0100
Fig. 7D	One way ANOVA	Ctrl vs. ATRA (MARS)	0.0004
		ATRA vs. ATRA+AHT (MARS)	0.9998
	One way ANOVA	Ctrl vs. ATRA (N-Hcy)	0.0083
		ATRA vs. ATRA+AHT (N-Hcy)	0.0097
Fig. 7I	One way ANOVA	ATRA-AHT- vs. ATRA+AHT- (SOD1)	0.0006
		ATRA+AHT- vs. ATRA+AHT+ (SOD1)	0.0019
	One way ANOVA	ATRA-AHT- vs. ATRA+AHT- (SOD2)	0.0024
		ATRA+AHT- vs. ATRA+AHT+ (SOD2)	0.0597
Fig. 7K	One way ANOVA	Ctrl vs. ATRA	0.0002
		ATRA vs. ATRA+AHT	0.0017
		ATRA vs. ATRA+NAC	0.0002
Fig. 7L	One way ANOVA	Ctrl vs. ATRA	<0.0001
		ATRA vs. ATRA+AHT	<0.0001
		ATRA vs. ATRA+NAC	<0.0001
Fig. 7M	One way ANOVA	Ctrl vs. ATRA (E8.5)	<0.0001
		ATRA vs. ATRA+AHT (E8.5)	0.0001
		ATRA vs. ATRA+NAC (E8.5)	0.0012
	One way ANOVA	Ctrl vs. ATRA (E10)	<0.0001
		ATRA vs. ATRA+AHT (E10)	0.0009
		ATRA vs. ATRA+NAC (E10)	0.0004
Fig. 7Q	One way ANOVA	Ctrl vs. ATRA	<0.0001

		ATRA vs. ATRA+AHT	0.0001
		ATRA vs. ATRA+NAC	0.0002

Appendix Figures

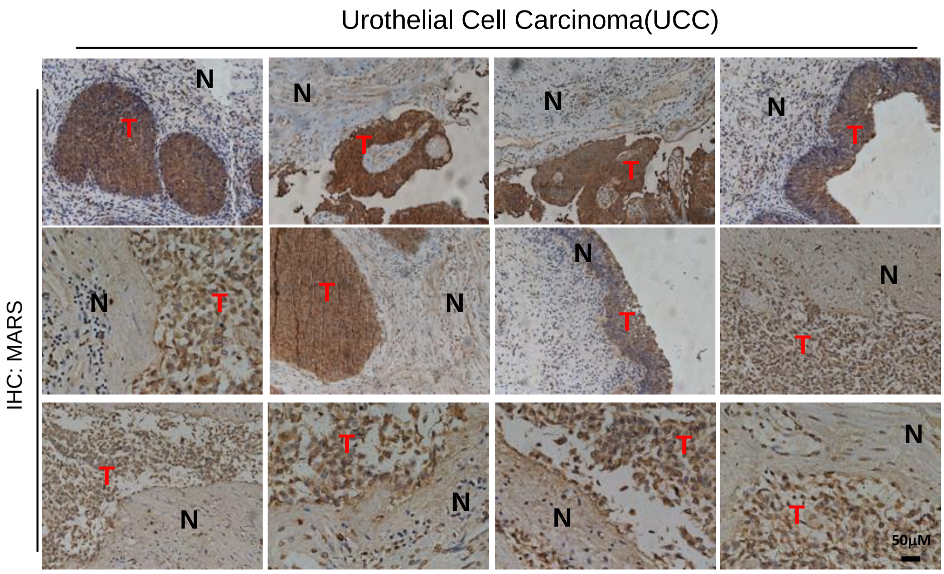

Appendix Figure S1. The expression of MARS in colorectal cancer (T) and adjacent non-tumor tissue (N) was detected (n=12, scale bar 50 μm).

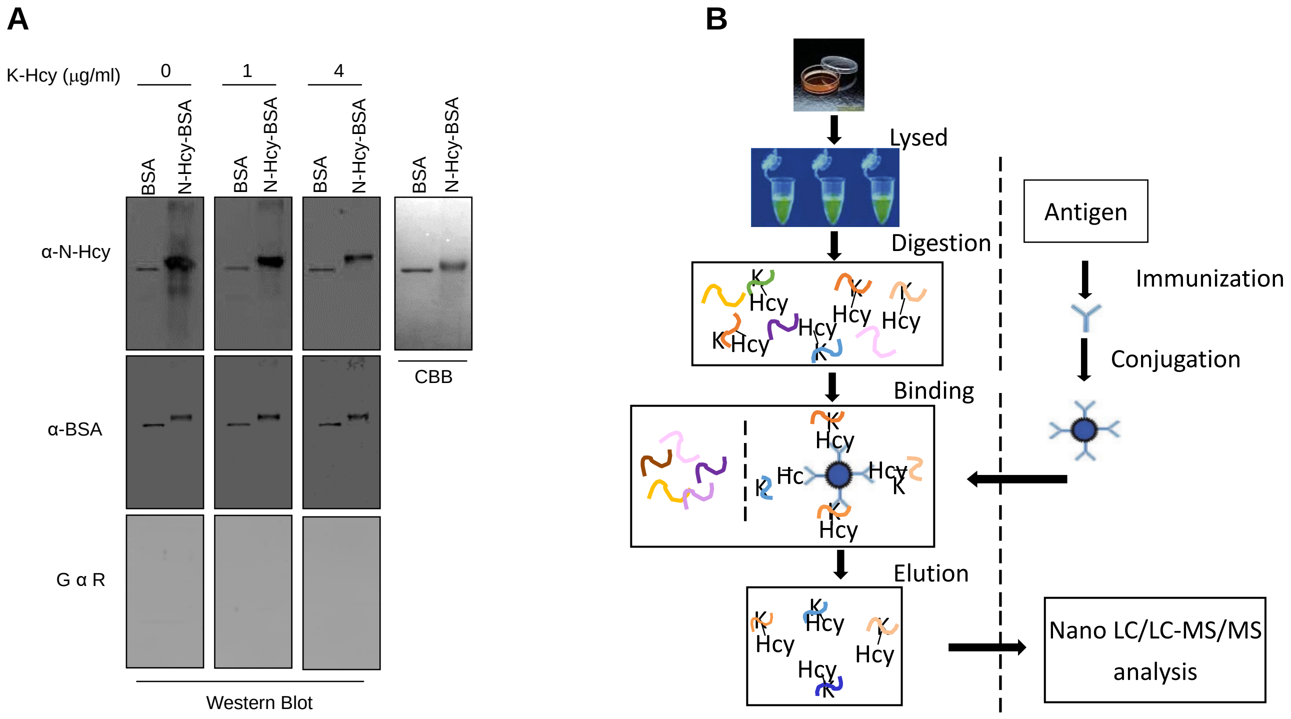

Appendix Figure S2. Strategy for the cell-wide proteomic survey for N-Hcy substrates (**A**) Verification of non-commercially produced α-N-Hcy antibody. The homocysteinylation levels of BSA and N-Hcy-modified BSA were detected with α-N-Hcy antibody produced in our lab in the presence of 0, 1, or 4 µg/mL Hcy-modified lysine (K-Hcy as a competitor). Proteins were resolved in 10% SDS-PAGE and transferred to nitrocellulose membranes. After blocking by peptone, the membranes were incubated with 1:1,000 antiserum. BSA signals were detected by western blot and used as a control. "G α R" indicates western blot analysis using secondary antibodies (Goat anti Rabbit) alone. (**B**) Flowchart depicting the N-Hcy substrate detection strategy. The proteome of HEK293T cells was digested with trypsin, and the resulting peptides were enriched for N-homocysteinylation-modified peptides. The eluted N-homocysteinylated peptides were analysed by LC-MS/MS. Coloured lines represent tryptic peptides from the HEK293T cell proteome; red dots on peptides indicate N-homocysteinylated lysines.

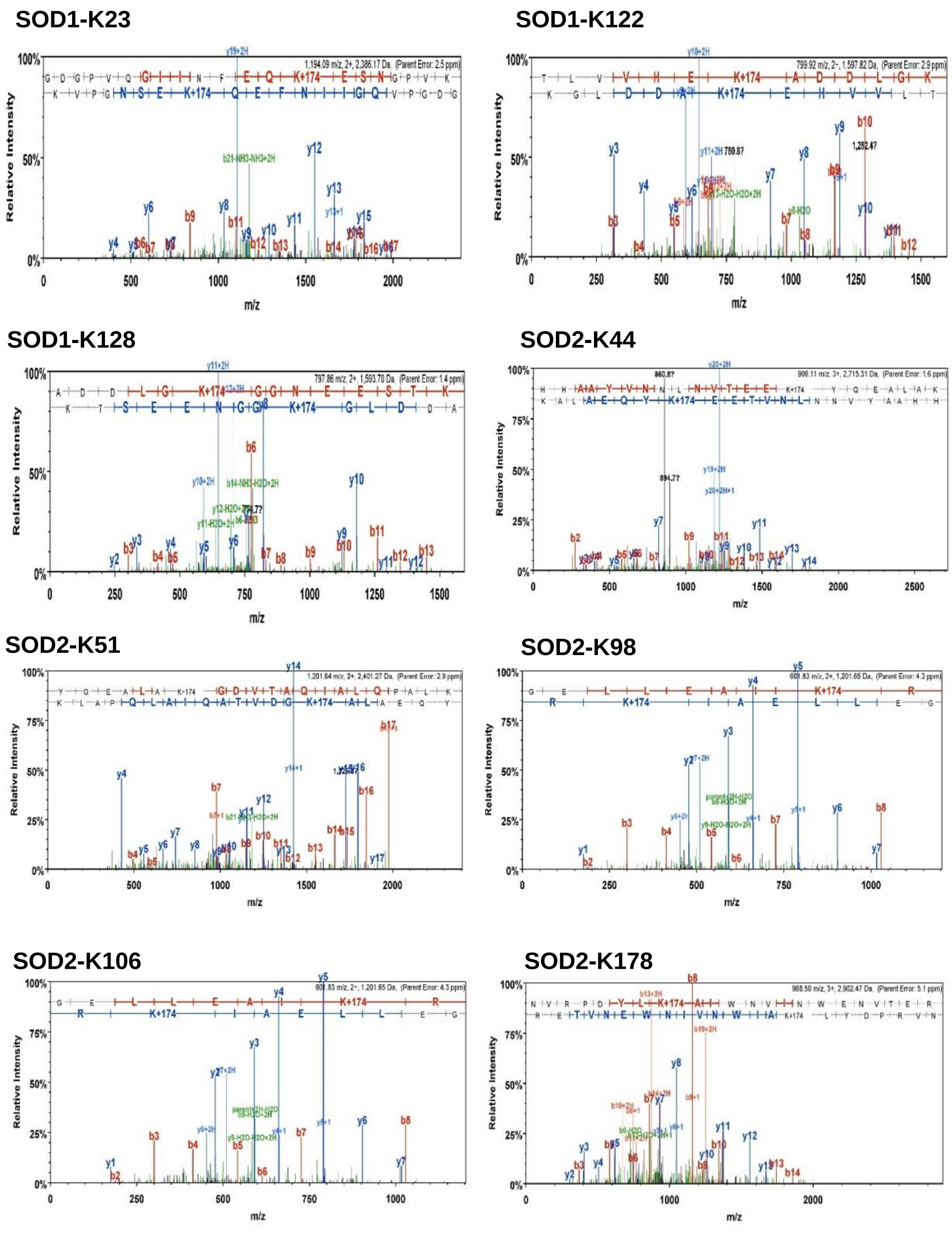

Appendix Figure S3. MS/MS spectra of lysine residues which are N-Hcy modified that led to the identification of K23, K122 and K128 of SOD1, K44, K51, K98, K106 and K178 of SOD2 were N-Hcy modified are shown.

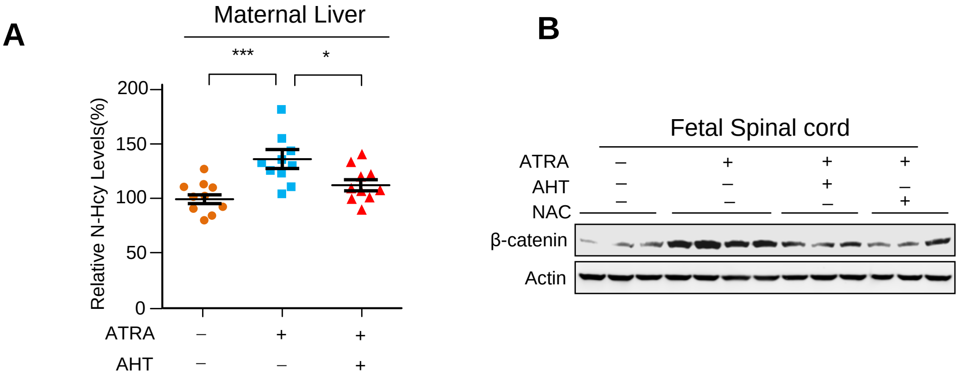

Appendix Figure S4. AHT decrease N-Hcy and β-catenin levels (**A**) N-Hcy levels of maternal livers were detected by Western blot. The results were quantified by Image J and normalized to the untreated group (Data were from 3 Western blot experiments). (**B**) Fetal brains were randomly selected and homogenized at E18.5 from litters of untreated, ATRA-treated, ATRA plus AHT and ATRA plus NAC co-treated pregnant rats. β-catenin levels were determined by Western blot. *P ⩽ 0.05; **P ⩽ 0.01; ***P ⩽ 0.001. One-way ANOVA with Dunnett's correction was used for multiple comparisons.
