# Supplementary material for: Inhibiting MARSs reduces hyperhomocysteinemia‐associated neural tube and congenital heart defects
Source: EMBO Mol Med. 2020 Jan 31;12(3):e9469. doi: 10.15252/emmm.201809469 (PMC7059139; doi:10.15252/emmm.201809469)
Supplement: Supplementary file 2 — Expanded View Figures PDF [file EMMM-12-e9469-s002.pdf]

Expanded View Figures

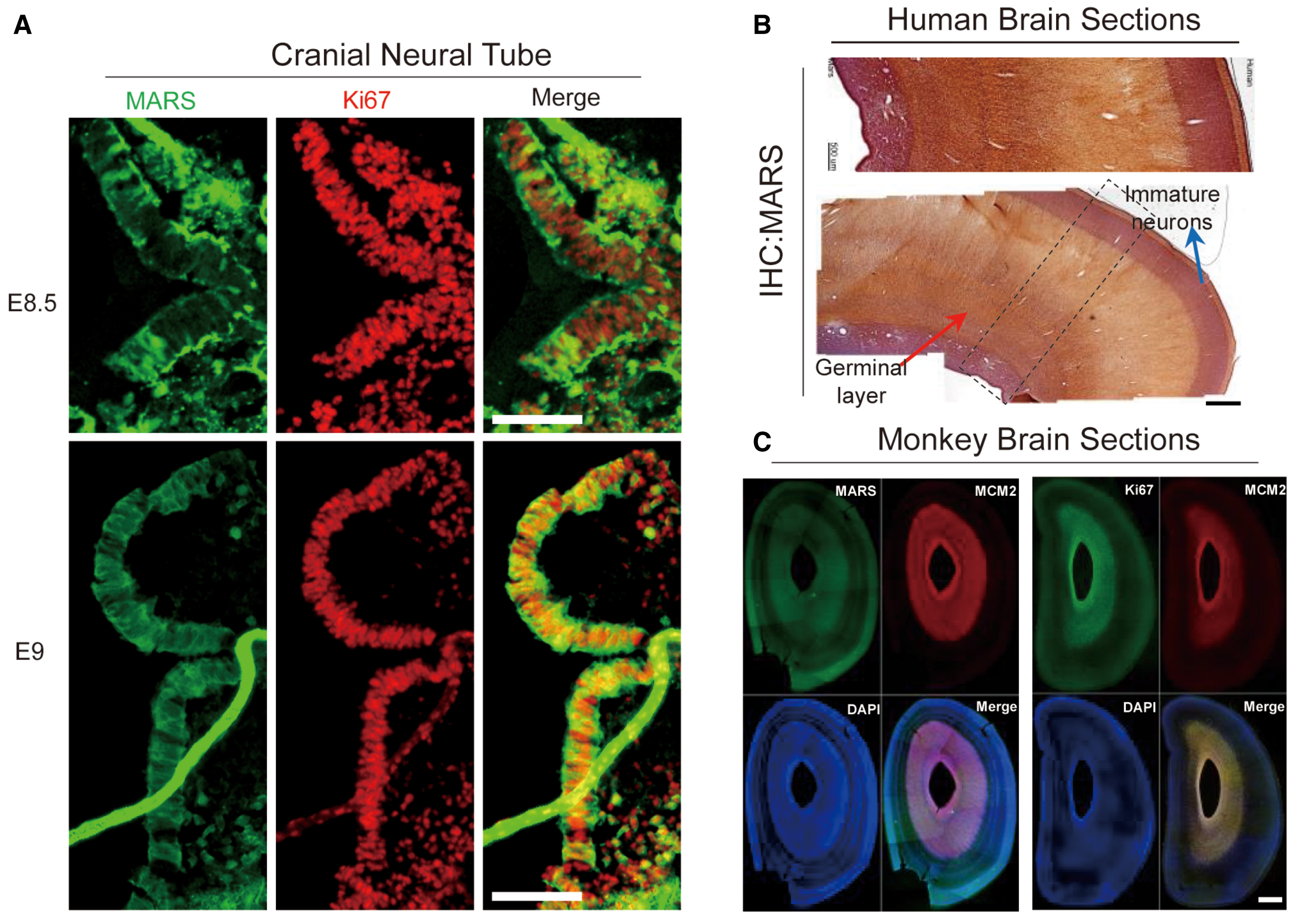

Figure EV1. Developing and proliferating cells have high MARS levels.

A Double IHC staining for MARS (green) and Ki67 (red) in brain sections at E8.5 and E9. Scale bar: 150 μm.

B MARS2 expression was detected in human brain sections (n = 3) by IHC. Local large magnification is shown as framed by dashed lines. Red arrow indicates germinal layer, blue indicates immature neurons. Scale bar: 1 mm.

C (Left) Proliferation markers Ki67 and MCM2 were double-immunostained in coronal brain sections of the E80 monkey brain. (Right) Double-stained MARS and MCM2. Scale bar: 1 mm.

© 2020 The Authors

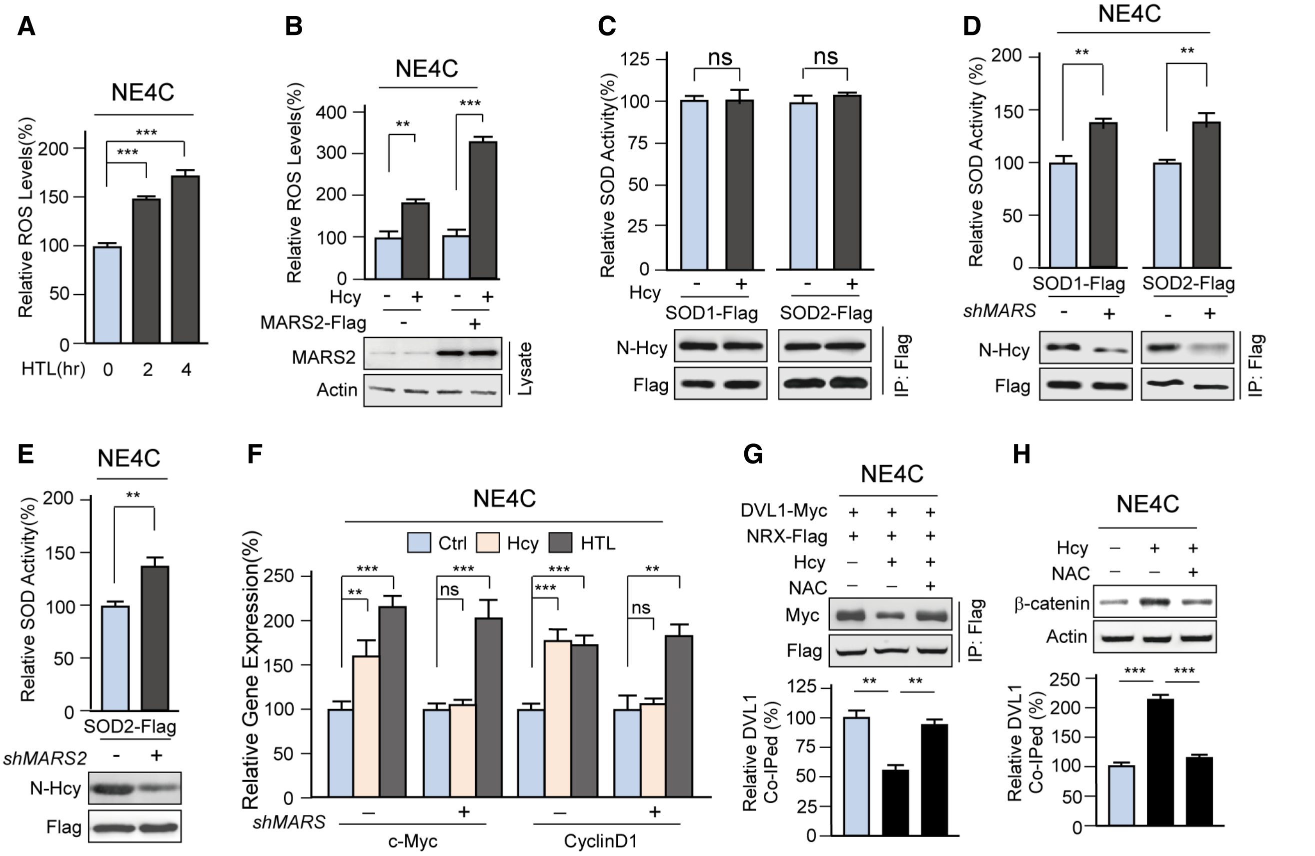

Figure EV2. MARS regulates ROS and Wnt/β-catenin signalling by modulating SOD N-Hcy.

- A Cellular superoxide levels were determined (n = 4) for NE4C cells treated with 20 μM HTL for different lengths of time. Superoxide levels were normalized to those of untreated NE4C cells.
- B Superoxide levels in NE4C cells (n = 3) were determined in the absence and presence of 20 μM Hcy in the culture media in control and MARS2-over-expressing cells (normalized to untreated cells).
- C N-homocysteinylation levels and specific activities of recombinant SOD1 and SOD2 were determined (n = 3) with and without incubation with 20 μM Hcy in tubes (*in vitro*). SOD enzymes were separated from the reaction mixture following the treatment. The specific activities of the SOD1 and SOD2 mutants were normalized to those of untreated SOD1 and SOD2, respectively (n = 4).
- D Flag-tagged SOD1 and SOD2 were each over-expressed in untreated and MARS knockdown NE4C cells. After affinity purification, N-homocysteinylation levels and the relative specific activities of purified SOD1 and SOD2 were determined relative to those in untreated NE4C cells (n = 4).
- E Flag-tagged SOD2 was over-expressed in untreated and MARS2 knockdown NE4C cells. After affinity purification, N-homocysteinylation levels and the relative specific activities of purified SOD2 were determined (n = 4).
- F Untreated and MARS/MARS2 knockdown NE4C cells were treated with either homocysteine (20 μM) or HTL (10 μM), and the gene expression of c-Myc and cyclin D1 was detected 6 h after the start of the respective treatment by real-time PCR. Error bars indicate SEM (n = 4).
- G NRX-Flag and DVL1-Myc were co-transfected in NE4C cells. Cells were treated with Hcy and NAC as indicated. The relative DVL1 levels that co-immunoprecipitated with NRX-Flag were determined and quantified (n = 3).
- H Levels of β-catenin in response to Hcy and NAC treatment in NE4C cells were detected by Western blot (n = 3). Representative Western blots are shown. The average intensities of quantified bands in untreated groups were set at 100%.

Data Information: Data are presented as the means ± SEM and were compared using an unpaired Student's t test. ^{ns}not significant, *$P \leq 0.05$; **$P \leq 0.01$; ***$P \leq 0.001$. One-way ANOVA with Dunnett's correction was used for multiple comparisons.

 © 2020 The Authors

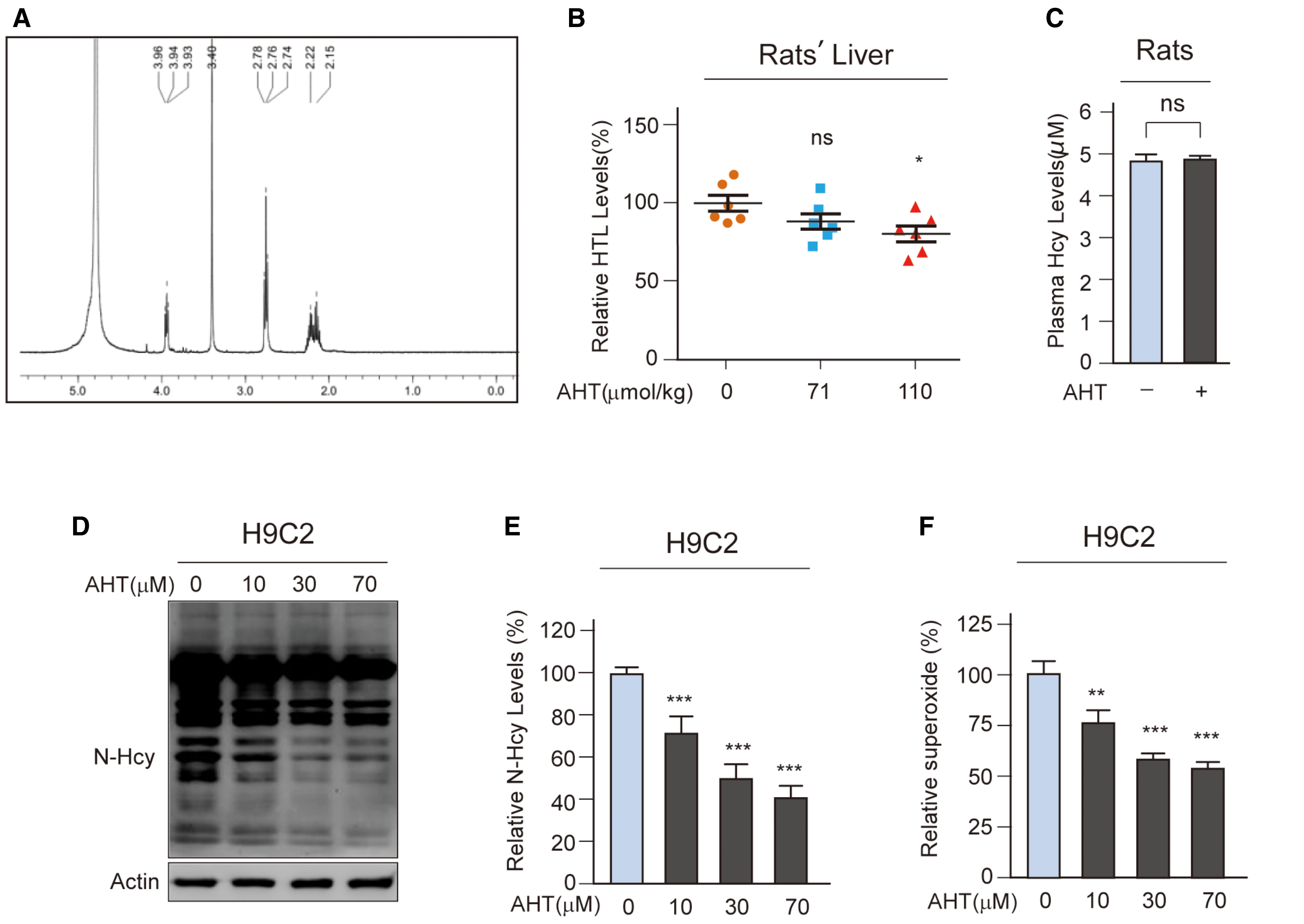

Figure EV3. AHT decreased protein N-Hcy levels.

A Nuclear magnetic resonance (NMR) spectroscopic analysis was carried out to verify the success of synthesis. The ^{1}H NMR (D_2O) of AHT is shown: δ 3.94 (t, J = 8 Hz, 1 H), 3.40 (s, 2 H), 2.76 (t, J = 8 Hz, 2 H), 2.22–2.15 (m, 2 H) (Thomsen *et al*, 2013).

B AHT (71 or 110 μg/kg per day) was injected in SD rats for 6 days. Relative HTL levels in liver homogenates were compared between AHT-treated and untreated SD rats.

C Plasma Hcy levels in AHT-treated or untreated rats were determined by a biochemistry analyser (n = 4).

D, E H9C2 cells were cultured in DMEM with 10 μM Hcy and supplemented the indicated AHT levels; N-homocysteinylation levels were detected by Western blot. The data of N-Hcy level quantification relative to the untreated group are shown in (E) (n = 4).

F Superoxide levels in H9C2 cells (n = 4) were determined in the absence or presence of 10 μM Hcy in the culture media (normalized to untreated cells).

Data information: Data are presented as the means ± SEM and were compared using an unpaired Student's t test. ^{ns}not significant, *$P \le 0.05$; **$P \le 0.01$; ***$P \le 0.001$. One-way ANOVA with Dunnett's correction was used for multiple comparisons.

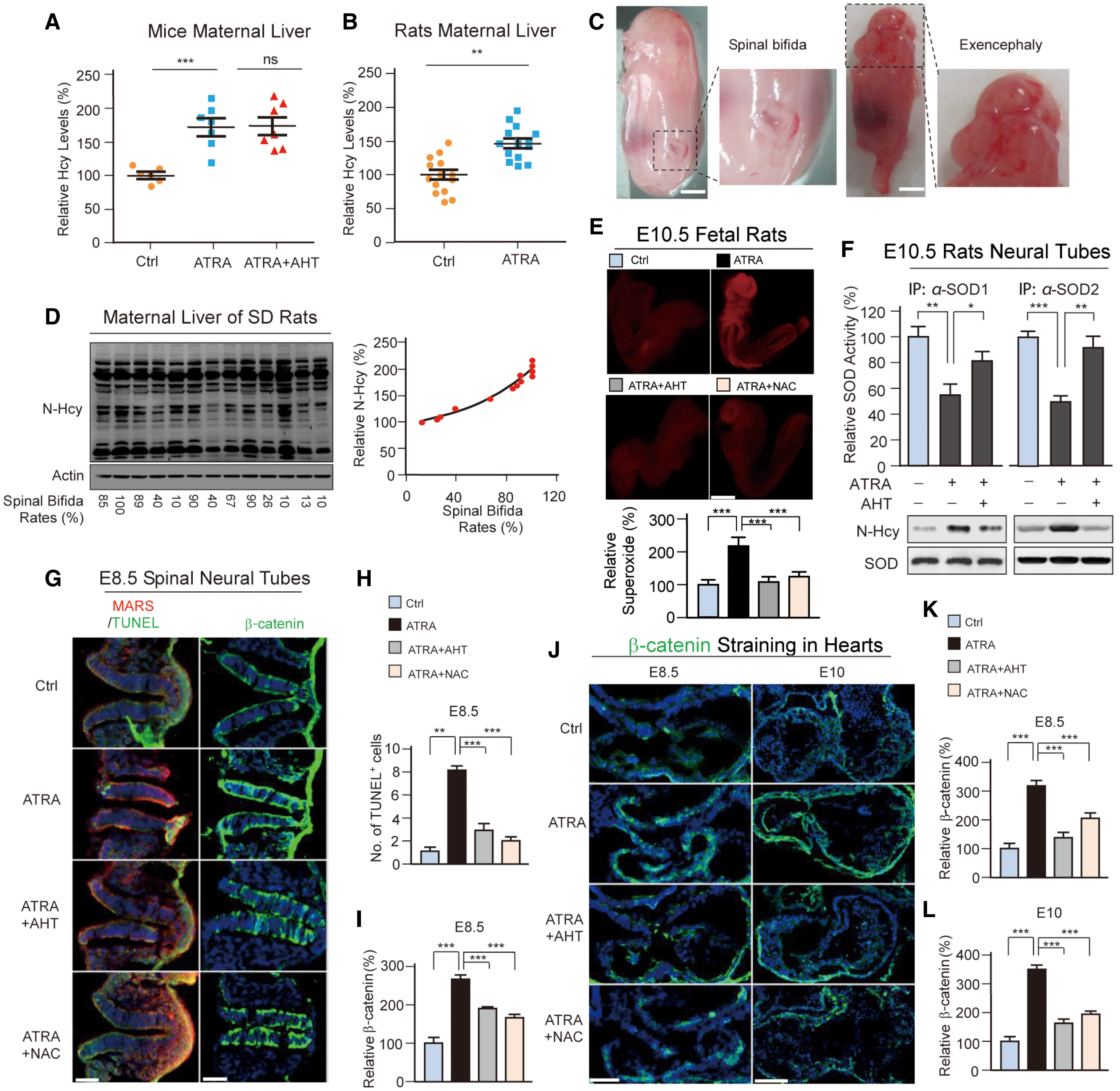

Figure EV4.

 © 2020 The Authors

Figure EV4. Targeting MARS to inhibit N-Hcy decreased the ROS, β-catenin and apoptosis levels.

A, B Hcy levels in maternal liver homogenates were detected in mice (A) and rats (B).

C ATRA-induced NTDs are exemplified by spina bifida (left) and exencephaly (right). Morphologies of E18.5 embryos from ATRA-treated rats were examined. Scale bar: 3 mm.

D Total protein N-Hcy levels in maternal rat liver homogenates were determined by Western blot and quantified in correlation to NTD prevalence in the corresponding foetuses.

E E10.5 foetal rats were subjected to DHE immunological staining to determine superoxide levels. Randomly selected foetal rats from litters of untreated, ATRA-, ATRA + AHT- and ATRA + NAC-treated pregnant rats were assayed (n = 3 each group). DHE fluorescence intensities are quantified at the bottom. Scale bar: 100 μm.

F SOD1 and SOD2 were immunoaffinity purified from E10.5 foetal rat neural tube tissues. The neural tube homogenates of each group were mixed from 6 embryos. N-Hcy levels and relative (to untreated) SOD activities were determined. Error bar indicates assay replicates (n = 4).

G MARS (red) elevation in E8.5 spinal neural tubes of ATRA-treated mice was associated with TUNEL (green, a) and β-catenin elevation. AHT and NAC treatment down-regulated TUNEL and β-catenin levels without affecting MARS levels. Scale bar: 300 μm.

H Quantification of TUNEL-positive cells in experiment (G). Three sections from three embryos were pooled for analysis.

I Quantification of β-catenin levels in E8.5 spinal neural tubes in (G). The mean value of β-catenin-positive cells/total cell number was set at 100%. Three sections from three embryos were pooled for analysis.

J IHC staining for β-catenin (green) in E8.5 and E10 heart sections (n = 6). Scale bar: 300 μm (E8.5), 600 μm (E10).

K, L Quantification of β-catenin levels in developing hearts at E8.5 (K) and E10 (L) from (J) (n = 6).

Data information: Data are presented as the mean ± SEM and were compared using an unpaired Student's t test. ^{ns}not significant, *$P \leq 0.05$, **$P \leq 0.01$, ***$P \leq 0.001$. One-way ANOVA with Dunnett's correction was used for multiple comparisons.
