# Supplementary material for: Inhibiting MARSs reduces hyperhomocysteinemia‐associated neural tube and congenital heart defects
Source: EMBO Mol Med. 2020 Jan 31;12(3):e9469. doi: 10.15252/emmm.201809469 (PMC7059139; doi:10.15252/emmm.201809469)
Supplement: Supplementary file 3 — Table EV1 [file EMMM-12-e9469-s003.docx]

**Table EV1. Site in SOD1 and SOD2 that are modifies by N-Hcy**

| SOD1 | K23 | K.GDGPVQGIINFEQK^#^ESNGPVK.V |
| --- | --- | --- |
|  | K122 | R.TLVVHEK^#^ADDLGK.G |
|  | K128 | K.ADDLGK^#^GGNEESTK.T |
| SOD2 | K44 | K.HHAAYVNNLNVTEEK^#^YQEALAK.G |
|  | K51 | K.YQEALAK^#^GDVTAQIALQPALK.F |
|  | K98 | K.GELLEAIK^#^R.D |
|  | K106 | R.DFGSFDK^#^FK.E |
|  | K178 | K.NVRPDYLK^#^AIWNVINWENVTER.Y |

Amino acid sequences of SOD1 and SOD2 with the lysine residues modified by N-Hcy (marked by #) are shown.
