# Supplementary material for: Inhibiting MARSs reduces hyperhomocysteinemia‐associated neural tube and congenital heart defects
Source: EMBO Mol Med. 2020 Jan 31;12(3):e9469. doi: 10.15252/emmm.201809469 (PMC7059139; doi:10.15252/emmm.201809469)
Supplement: Supplementary file 4 — Table EV2 [file EMMM-12-e9469-s004.docx]

**Table EV2. Rats embryonic parameters and NTDs incidence of ATRA exposure and AHT or NAC protection**

| E11.5(40) | Ctrl | ATRA | ATRA+AHT | ATRA+NAC |
| --- | --- | --- | --- | --- |
| No. Litters | 9 | 10 | 11 | 11 |
| No. implantation | 118 | 146 | 162 | 158 |
| No. Live fetuses | 118 | 136 | 158 | 153 |
| Resorption | 0(0.0%) | 10(6.8%) | 4(2.5%) | 5(3.2%) |
| Totle NTDs^a,b,c^ | 0 | 119 (87.5%) | 66(41.8%) | 87(56.9%) |
| Spinal^a,b,c^ | 0 | 114 | 64 | 86 |
| Cranial | 0 | 3 | 1 | 0 |
| Multiple | 0 | 2 | 1 | 1 |

^a,b,c^ Significant(p<0.05) when comparisons are made as follows

^a^ Ctrl vs. ATRA, ^b^ ATRA vs. ATRA+AHT, ^c^ ATRA vs. ATRA+NAC. Fisher's exact test
