# Supplementary material for: Inhibiting MARSs reduces hyperhomocysteinemia‐associated neural tube and congenital heart defects
Source: EMBO Mol Med. 2020 Jan 31;12(3):e9469. doi: 10.15252/emmm.201809469 (PMC7059139; doi:10.15252/emmm.201809469)

Figure 1

Fig.1C MARS

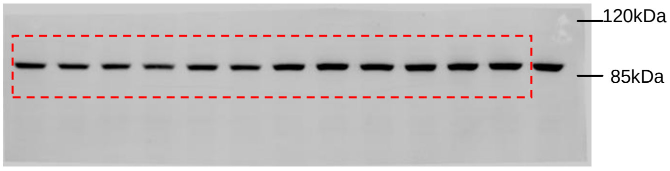

Fig.1C Actin

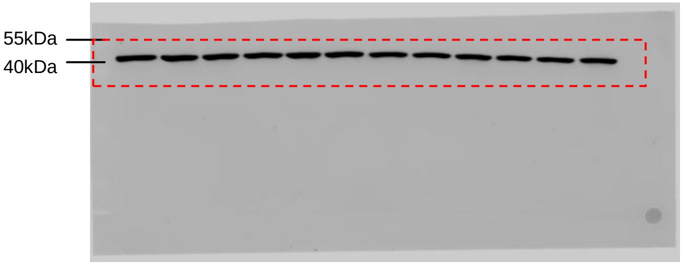

Fig.1E MARS2

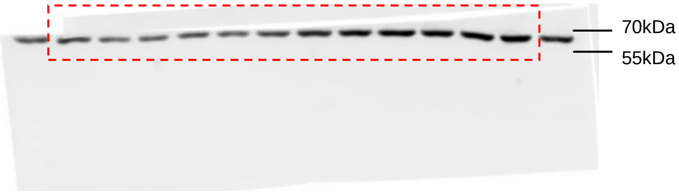

Fig.1E Actin

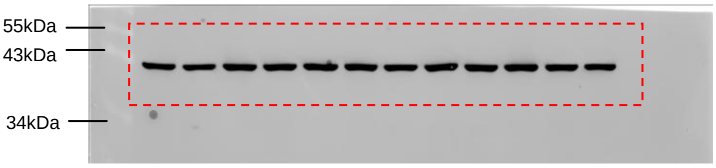

Fig.1G

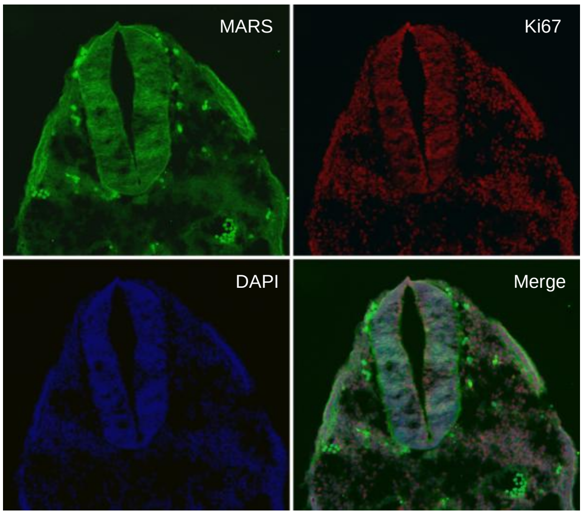

Fig.1H

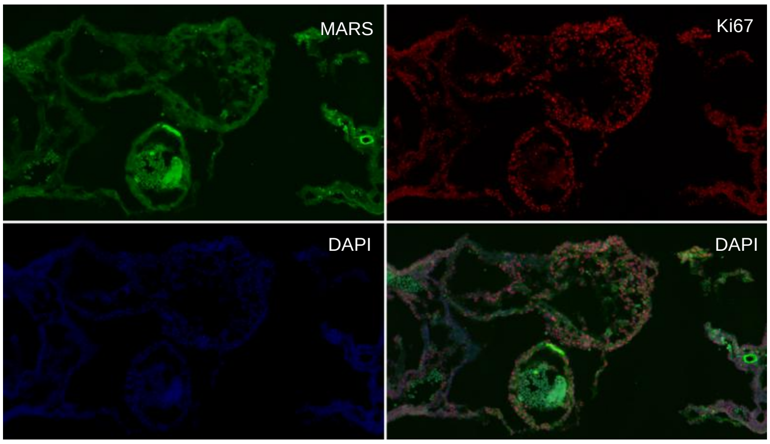

Fig.1J MARS

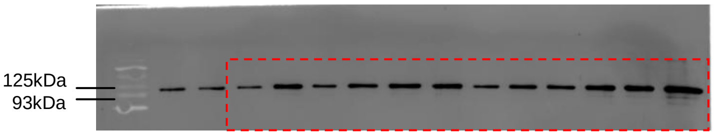

Fig.1J Actin

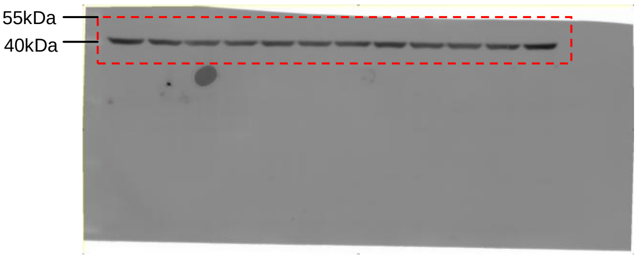

Fig.1O MARS

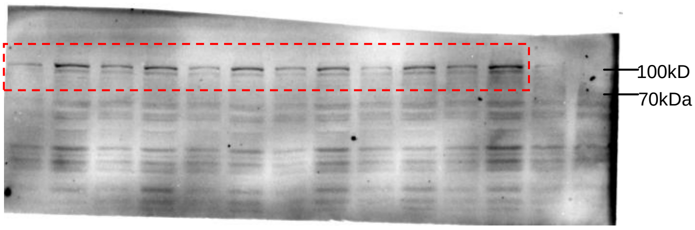

Fig.1O Actin

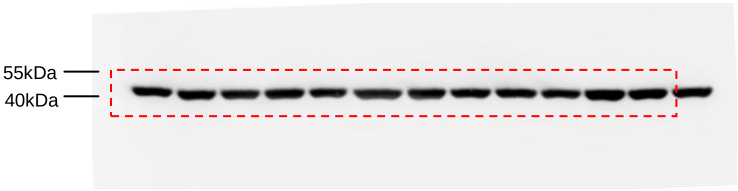

Supplement: Supplementary file 7 — Source Data for Figure 1 [file EMMM-12-e9469-s006.pdf]
