# Supplementary figures and images for: Inhibiting MARSs reduces hyperhomocysteinemia‐associated neural tube and congenital heart defects
Source: EMBO Mol Med. 2020 Jan 31;12(3):e9469. doi: 10.15252/emmm.201809469 (PMC7059139; doi:10.15252/emmm.201809469)

Figure 2

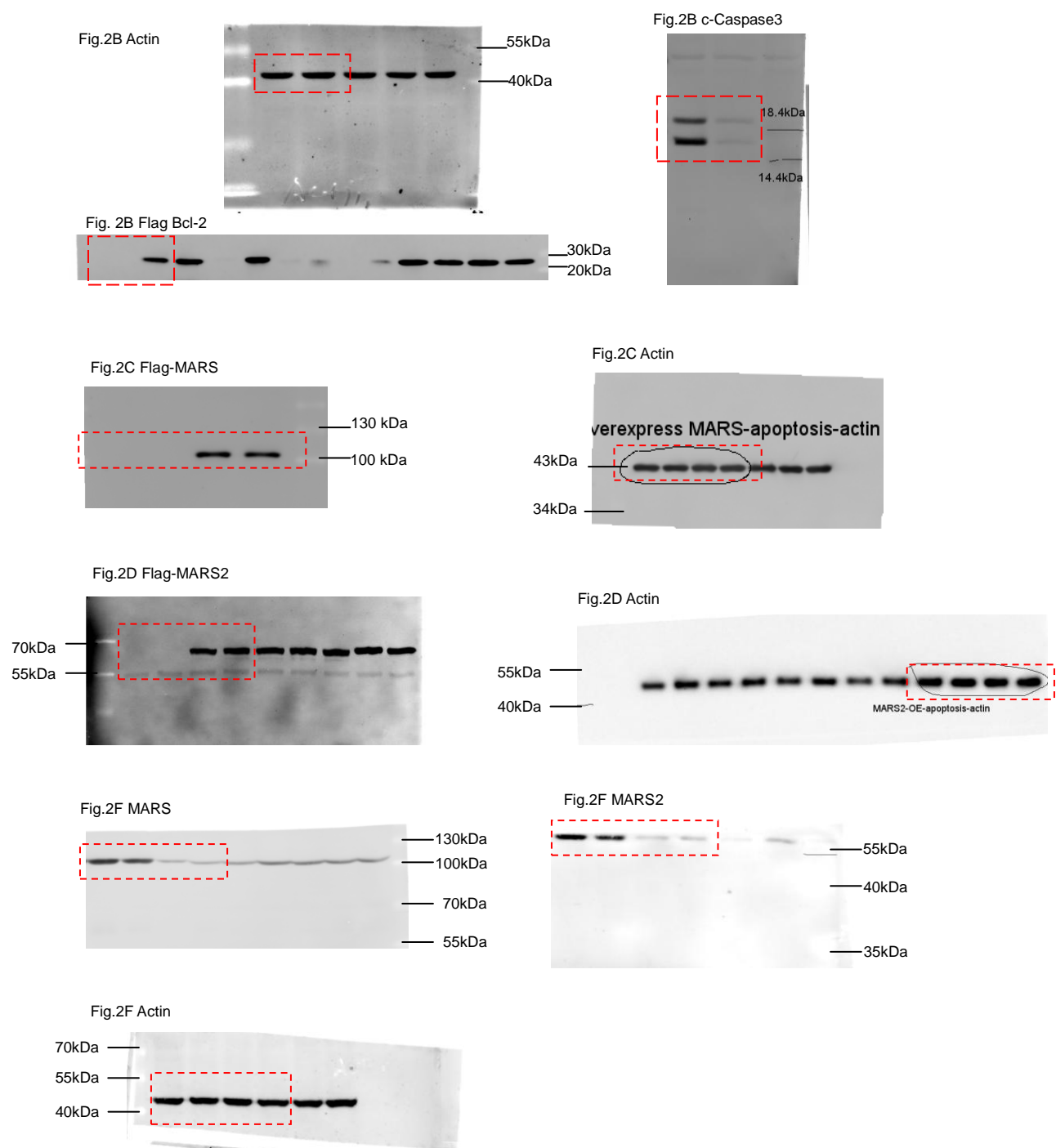

Supplement: Supplementary file 8 — Source Data for Figure 2 [file EMMM-12-e9469-s007.pdf]
