# Supplementary material for: Inhibiting MARSs reduces hyperhomocysteinemia‐associated neural tube and congenital heart defects
Source: EMBO Mol Med. 2020 Jan 31;12(3):e9469. doi: 10.15252/emmm.201809469 (PMC7059139; doi:10.15252/emmm.201809469)

Figure 3

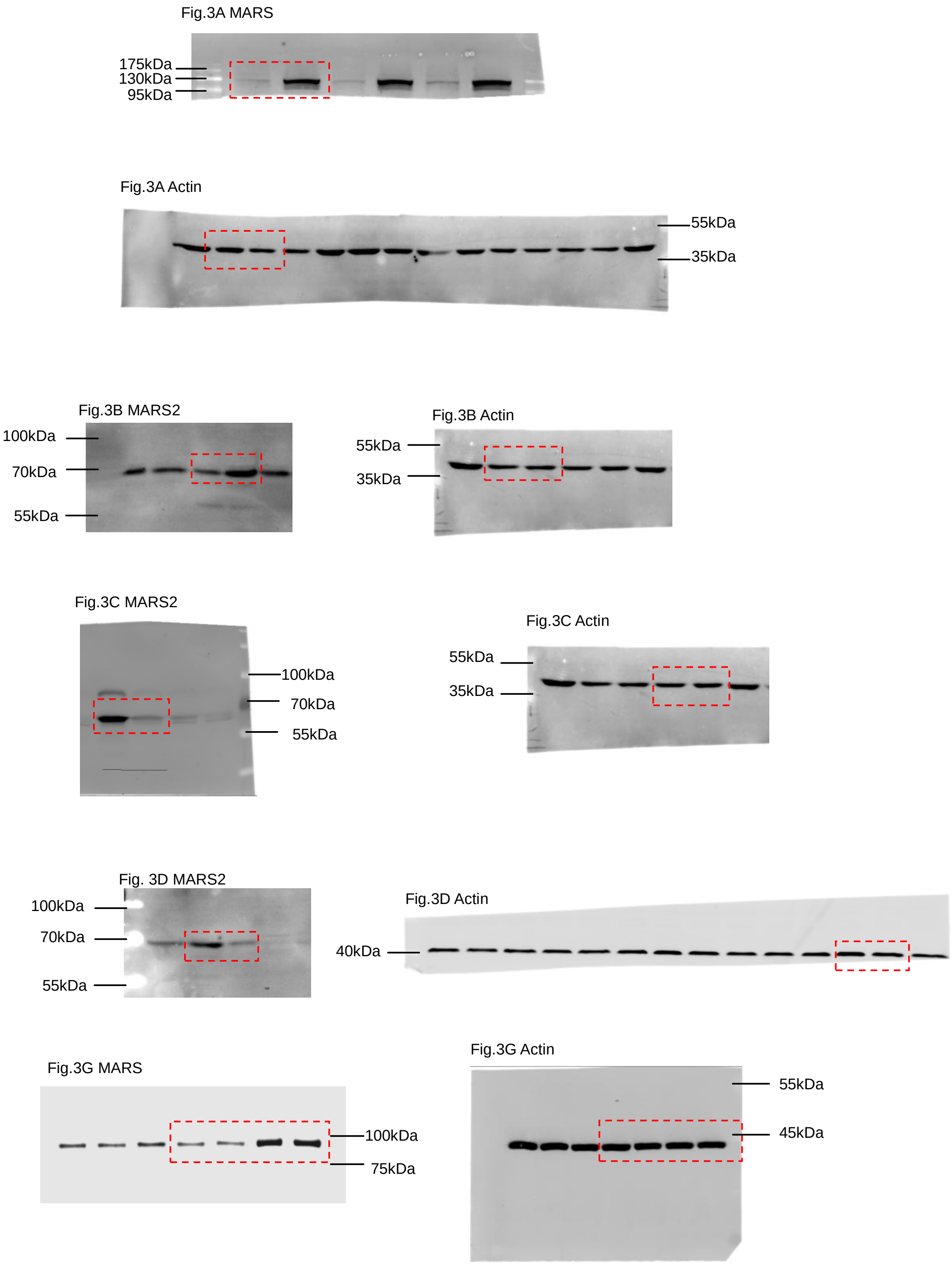
Fig.3A MARS
175kDa
130kDa
95kDa
Fig.3A Actin
55kDa
35kDa
Fig.3B MARS2
100kDa
70kDa
55kDa
Fig.3B Actin
55kDa
35kDa
Fig.3C MARS2
100kDa
70kDa
55kDa
Fig.3C Actin
55kDa
35kDa
Fig. 3D MARS2
100kDa
70kDa
55kDa
Fig.3D Actin
40kDa
Fig.3G MARS
100kDa
75kDa
Fig.3G Actin
55kDa
45kDa

Fig.3H N-Hcy

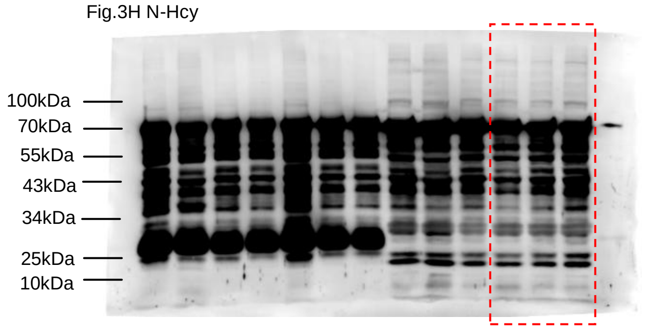

Fig.3H Actin,

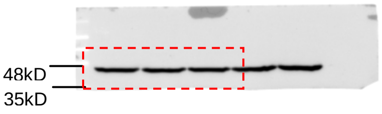

Fig.3I Actin

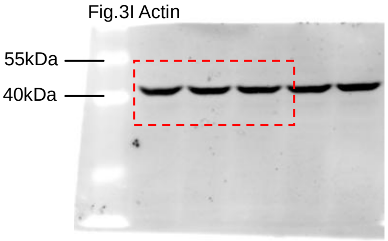

Fig.3I N-Hcy

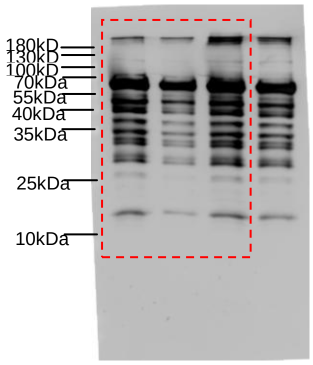

Fig.3I MARS ,

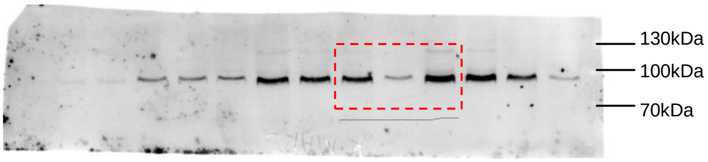

Fig.3J N-Hcy

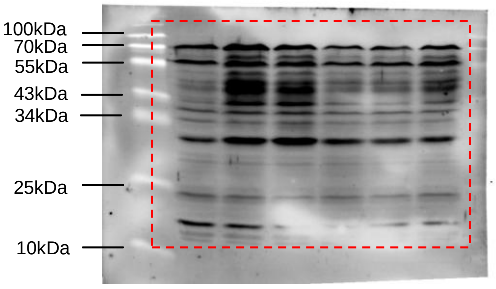

Fig.3J MARS

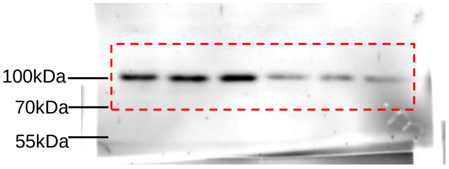

Fig.3J Actin

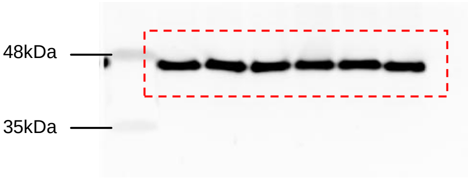

Fig.3J MARS2

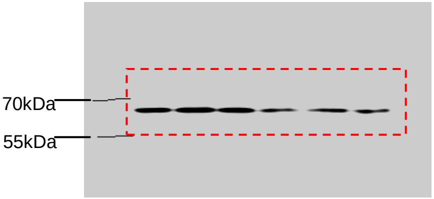

Supplement: Supplementary file 9 — Source Data for Figure 3 [file EMMM-12-e9469-s008.pdf]
