# Supplementary material for: Inhibiting MARSs reduces hyperhomocysteinemia‐associated neural tube and congenital heart defects
Source: EMBO Mol Med. 2020 Jan 31;12(3):e9469. doi: 10.15252/emmm.201809469 (PMC7059139; doi:10.15252/emmm.201809469)

Figure 4

Fig.4C Flag (SOD1)

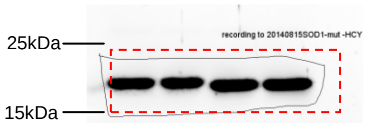

Fig.4C Flag (SOD2)

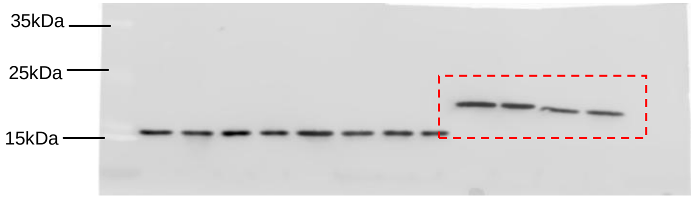

Fig.4C N-Hcy (SOD1)

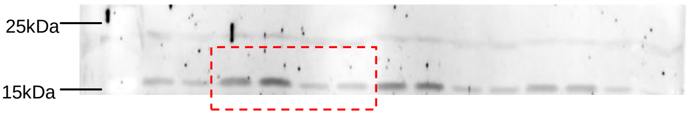

Fig.4C N-Hcy (SOD2)

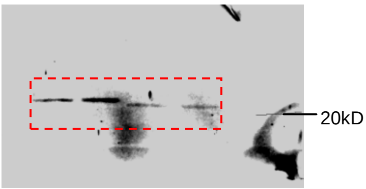

Fig.4E N-Hcy (SOD1)

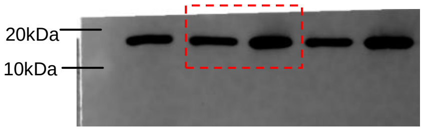

Fig.4 E Flag（SOD1）

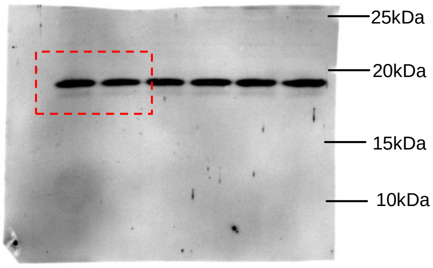

Fig 4E N-Hcy (SOD1)

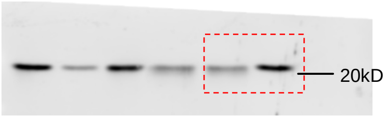

Fig.4E Flag (SOD2)

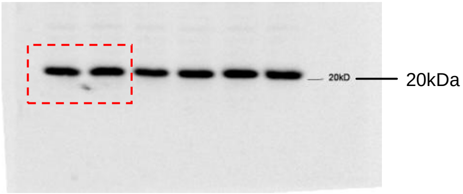

Fig.4F N-Hcy (SOD1)

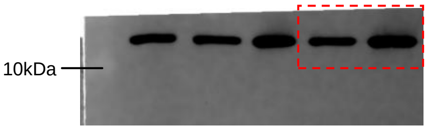

Fig.4F N-Hcy (SOD2)

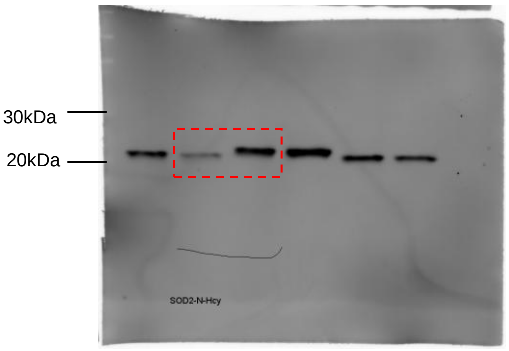

Fig.4G N-Hcy (SOD1)

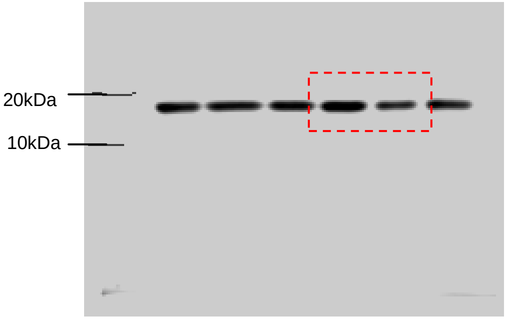

Fig 4G N-Hcy (SOD2)

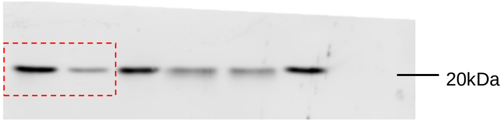

Fig.4F Flag (SOD1)

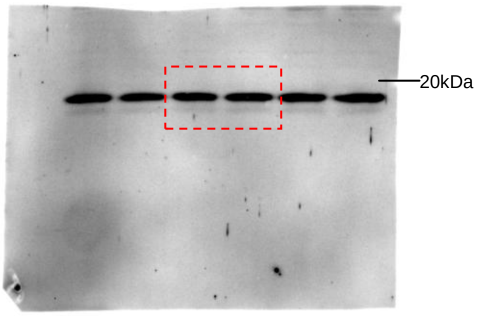

Fig.4F Flag (SOD2)

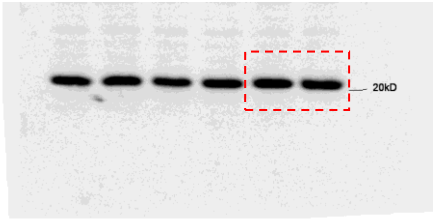

Fig.4G Flag (SOD1)

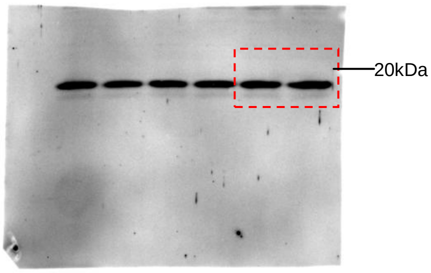

Fig.4 G Flag (SOD2)

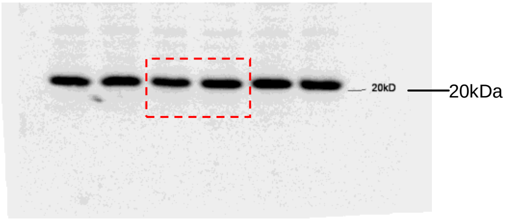

Supplement: Supplementary file 10 — Source Data for Figure 4 [file EMMM-12-e9469-s009.pdf]
