# Supplementary material for: Inhibiting MARSs reduces hyperhomocysteinemia‐associated neural tube and congenital heart defects
Source: EMBO Mol Med. 2020 Jan 31;12(3):e9469. doi: 10.15252/emmm.201809469 (PMC7059139; doi:10.15252/emmm.201809469)

Figure 5

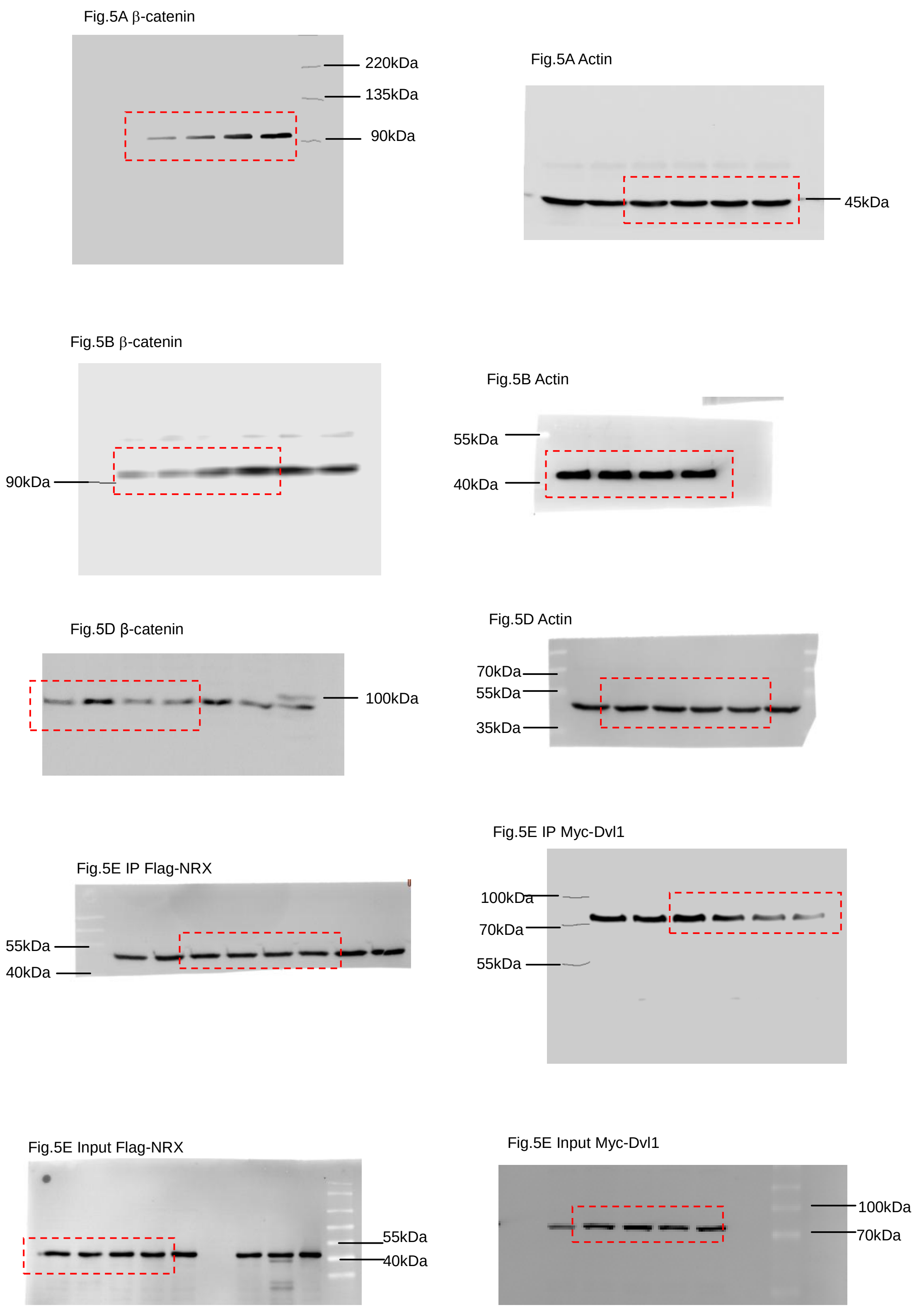

Fig.5A β-catenin
220kDa
135kDa
90kDa
Fig.5A Actin
45kDa
Fig.5B β-catenin
90kDa
Fig.5B Actin
55kDa
40kDa
Fig.5D β-catenin
100kDa
Fig.5D Actin
70kDa
55kDa
35kDa
Fig.5E IP Flag-NRX
55kDa
40kDa
Fig.5E IP Myc-Dvl1
100kDa
70kDa
55kDa
Fig.5E Input Flag-NRX
55kDa
40kDa
Fig.5E Input Myc-Dvl1
100kDa
70kDa

Fig.5F Input Flag-NRX

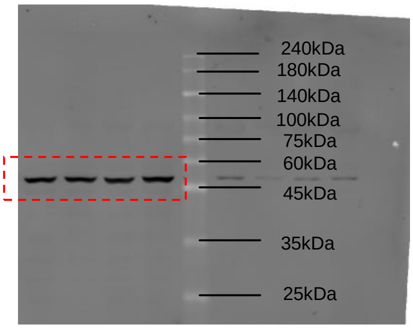

Fig.5F Input Myc-Dvl1

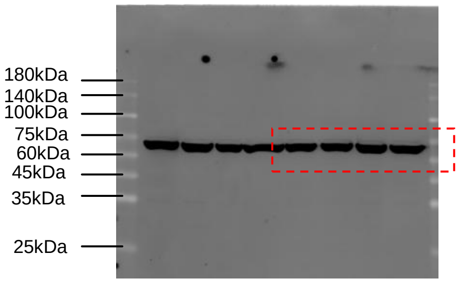

Fig.5F IP Flag-NRX

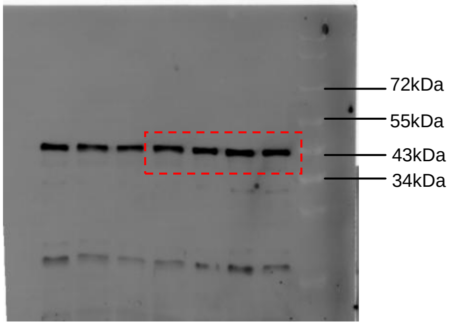

Fig.5F IP Myc-Dvl1

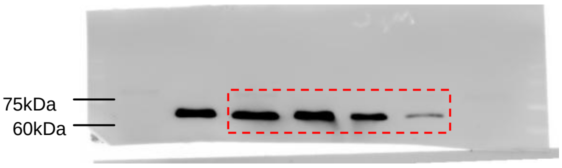

Fig.5G Input Flag-NRX

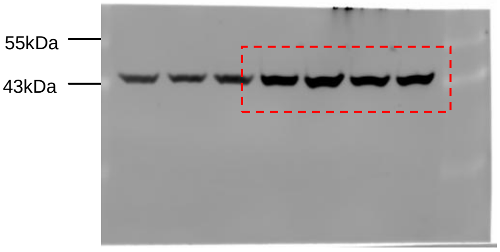

Fig.5G Input Myc-Dvl1

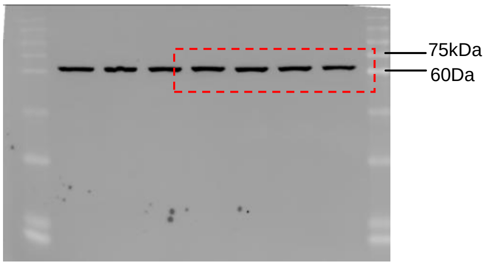

Fig.5G IP Flag-NRX

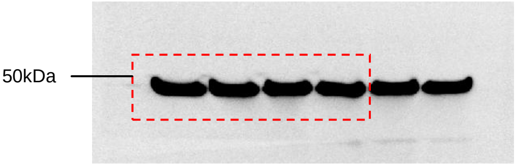

Fig.5g IP Myc-Dvl1

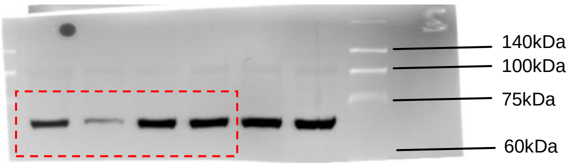

Fig.5H β-catenin

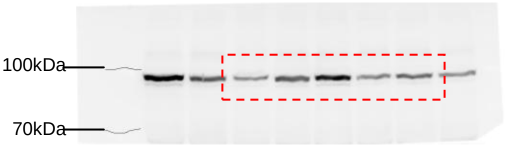

Fig.5H Flag

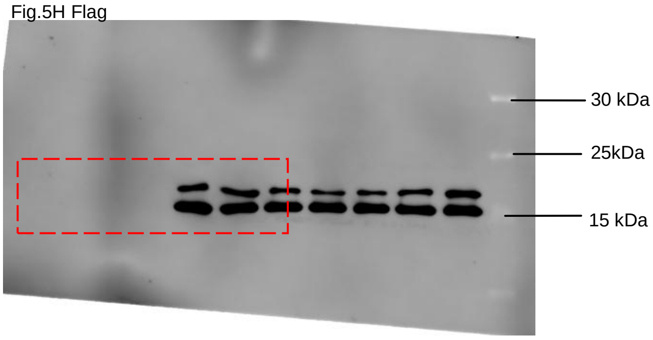

Fig.5H β-catenin

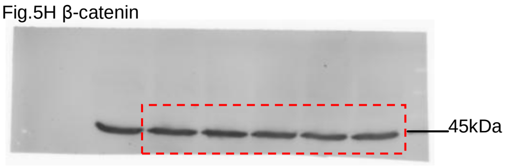

Fig.5I IP Flag

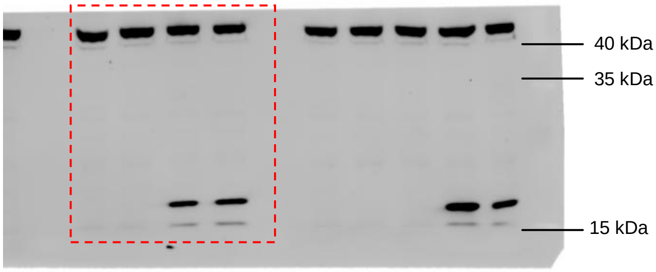

Fig.5I IP Myc-Dvl1

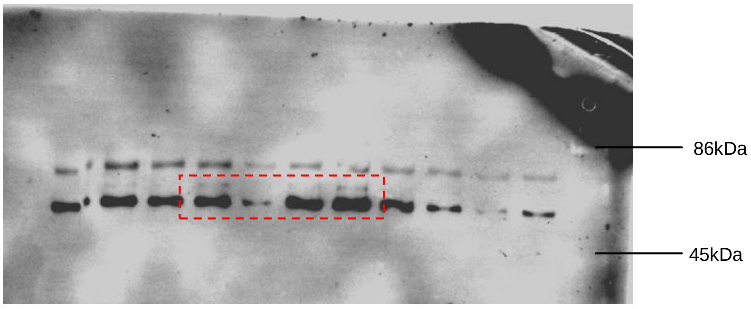

Fig.5I Input Flag

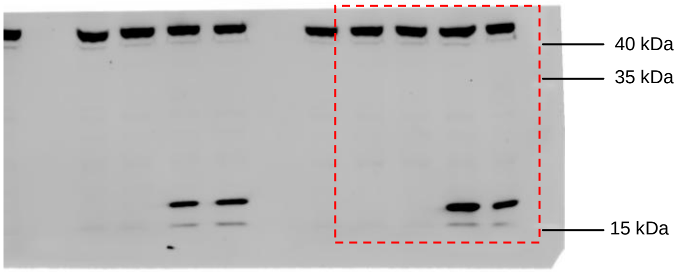

Fig.5I Input Myc-Dvl1

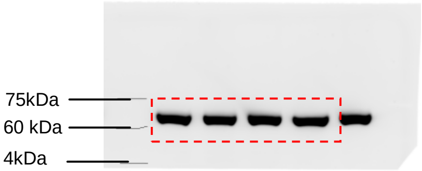

Supplement: Supplementary file 11 — Source Data for Figure 5 [file EMMM-12-e9469-s010.pdf]
