# Supplementary material for: Inhibiting MARSs reduces hyperhomocysteinemia‐associated neural tube and congenital heart defects
Source: EMBO Mol Med. 2020 Jan 31;12(3):e9469. doi: 10.15252/emmm.201809469 (PMC7059139; doi:10.15252/emmm.201809469)

Figure 6

Fig.6G   N-Hcy

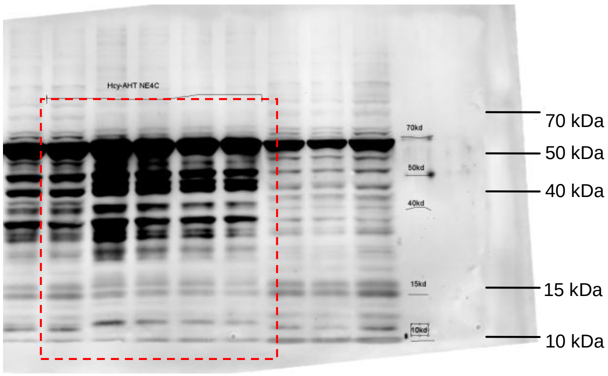

Fig.6G Actin

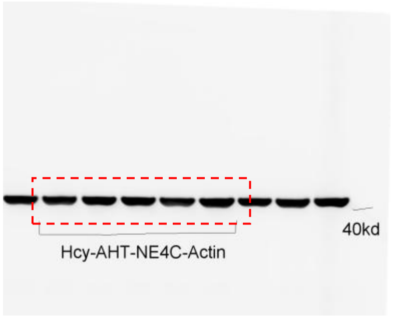

Fig.6K Actin (Hcy)   Fig.6K Actin (HTL)

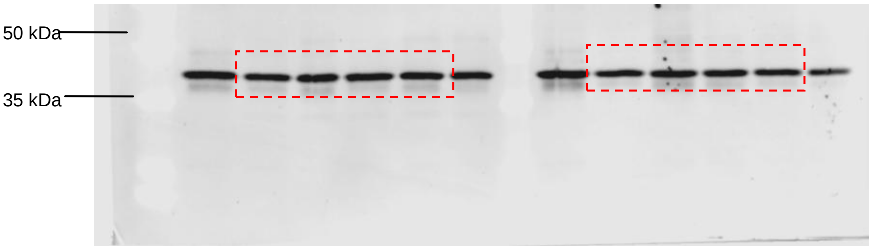

Fig.6K β-catenin (Hcy)

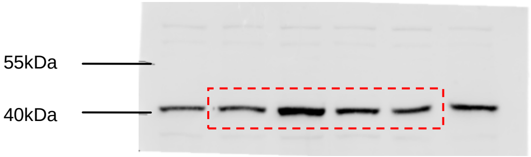

Fig.6Kβ-catenin (HTL)

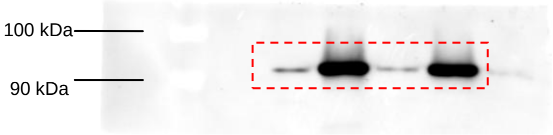

Supplement: Supplementary file 12 — Source Data for Figure 6 [file EMMM-12-e9469-s011.pdf]
