# Supplementary material for: Inhibiting MARSs reduces hyperhomocysteinemia‐associated neural tube and congenital heart defects
Source: EMBO Mol Med. 2020 Jan 31;12(3):e9469. doi: 10.15252/emmm.201809469 (PMC7059139; doi:10.15252/emmm.201809469)

Figure 7

Fig7A Ctrl

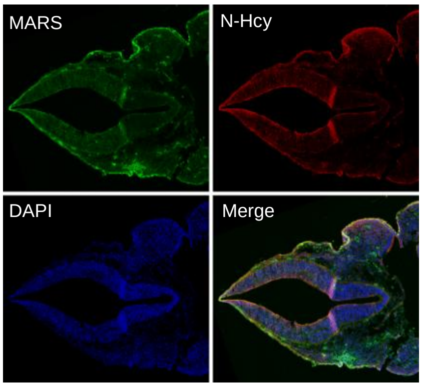

Fig7A ATRA

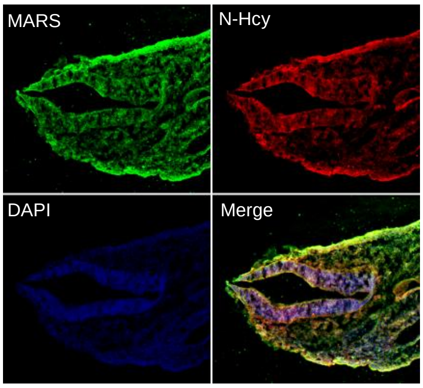

Fig7A ATRA + AHT

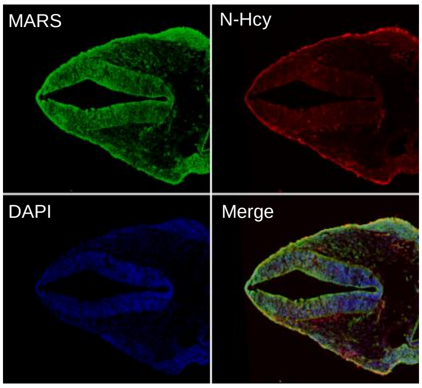

Fig7C Ctrl

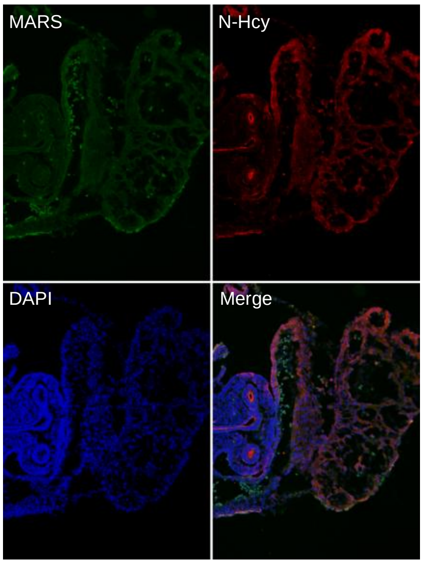

Fig7A ATRA

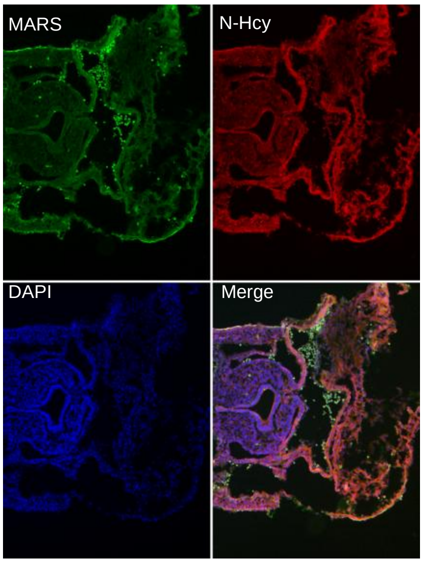

Fig7A ATRA + AHT

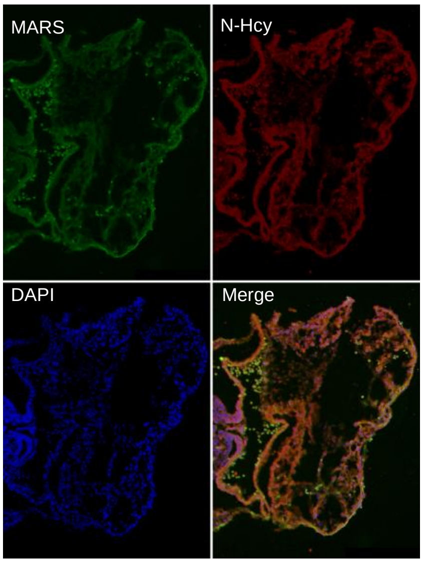

Fig.7H N-Hcy

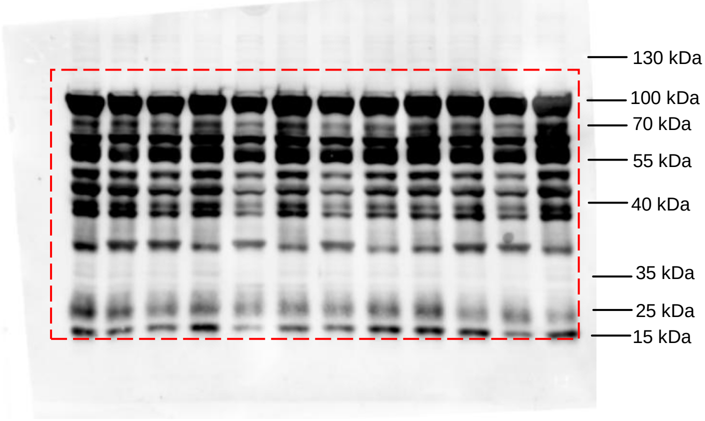

Fig.7H Actin

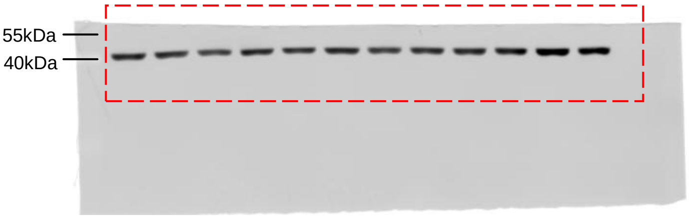

Fig.7I SOD1 N-Hcy

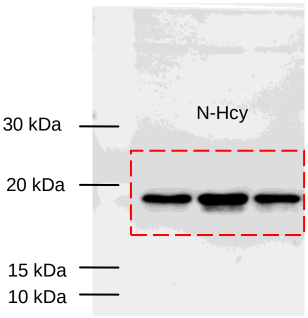

Fig.7I SOD1

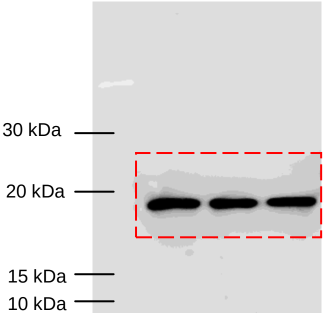

Fig.7I SOD2 N-Hcy

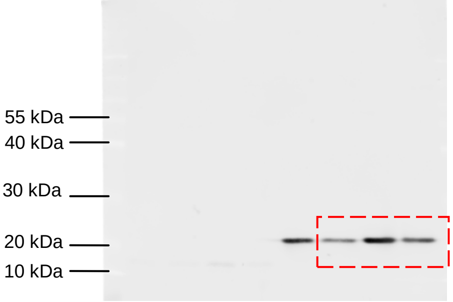

Fig.7I SOD2

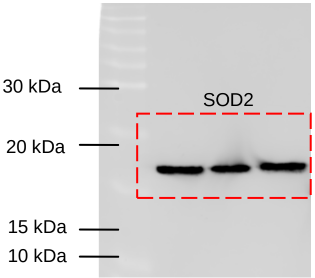

Fig. 7J-b-Ctrl

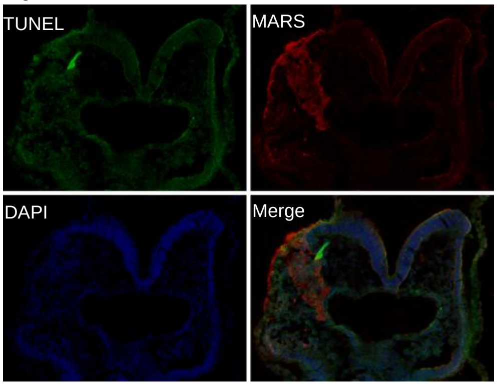

Fig. 7J-b-ATRA

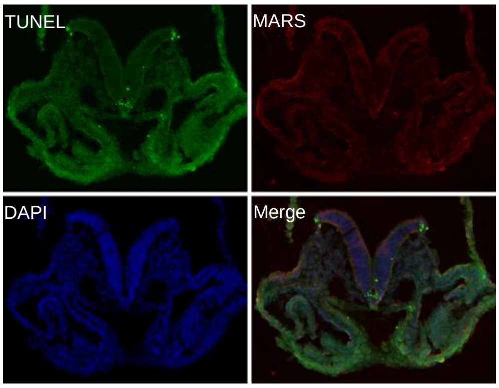

Fig. 7J-b-ATRA+AHT

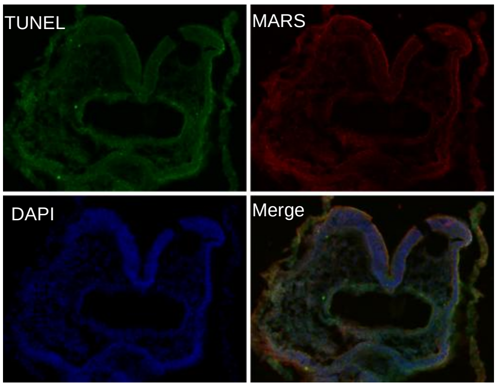

Fig. 7J-b-ATRA+NAC

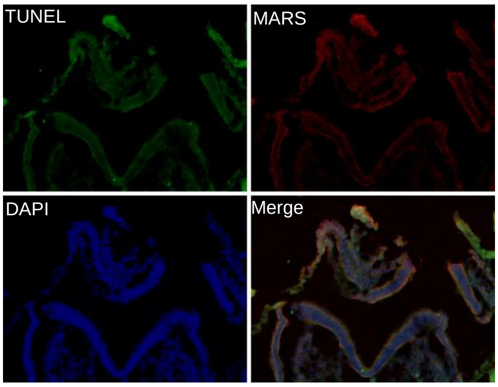

Fig. 7J-c-Ctrl

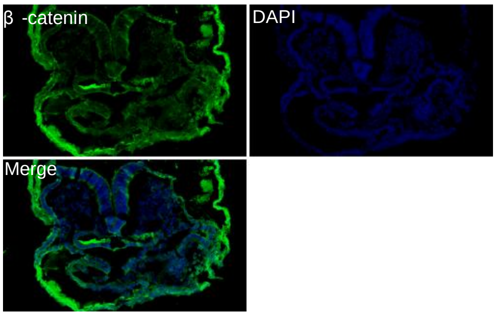

Fig. 7J-c-ATRA

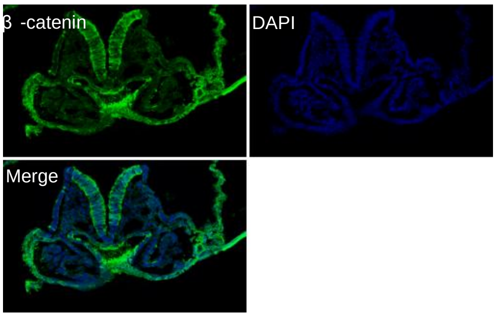

Fig. 7J-c-ATRA+AHT

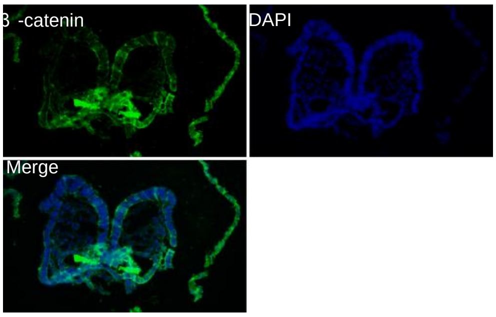

Fig. 7J-c-ATRA+NAC

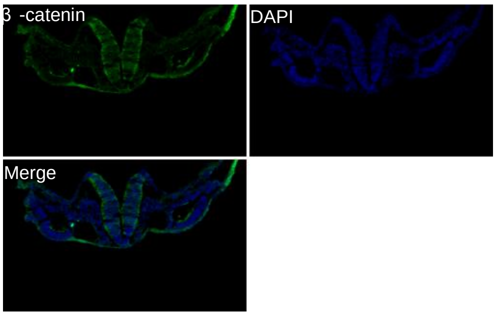

Fig. 7J-d-Ctrl

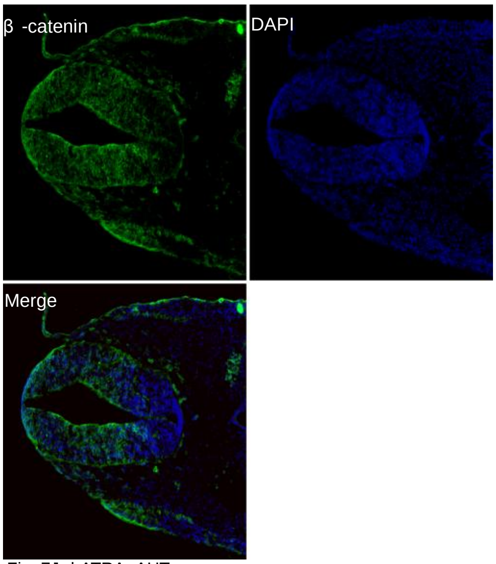

Fig. 7J-d-ATRA

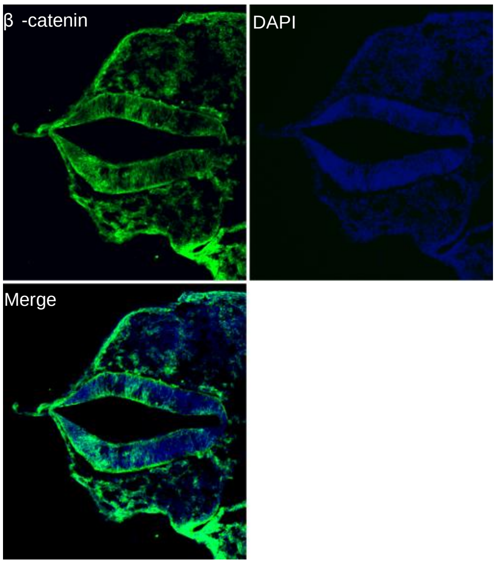

Fig. 7J-d-ATRA+AHT

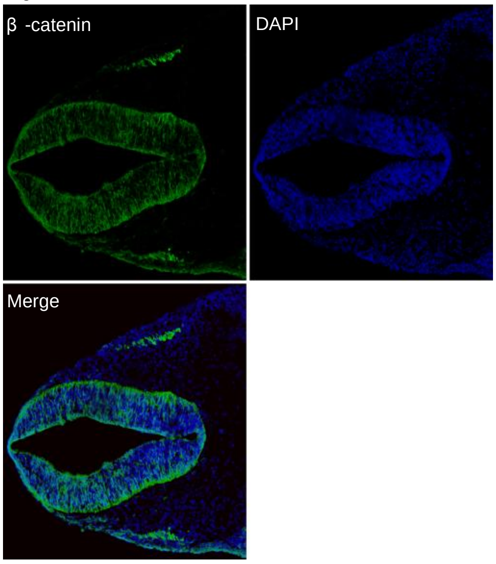

Fig. 7J-d-ATRA+NAC

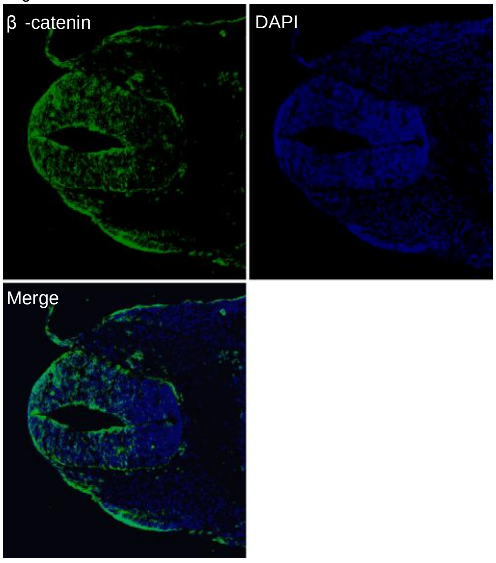

Fig.7O N-Hcy

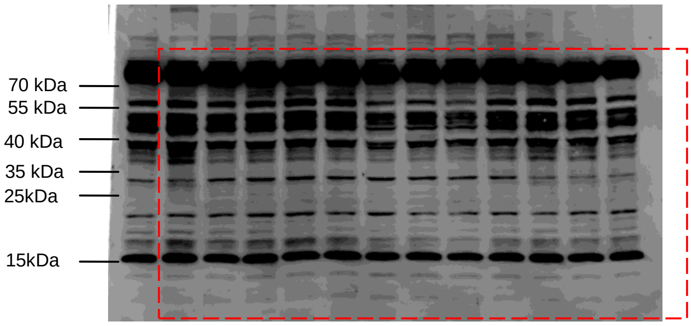

Fig.7O Actin

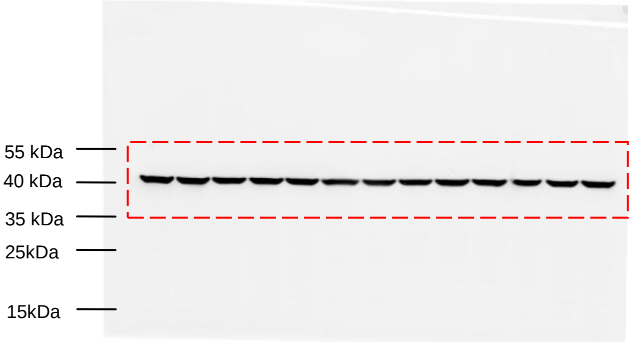

Supplement: Supplementary file 13 — Source Data for Figure 7 [file EMMM-12-e9469-s012.pdf]
